# The Evolution and Origin of Allotetraploid *Aegilops geniculata* Revealed by the Homoeolog‐Resolved Genome Assembly

**DOI:** 10.1111/pbi.70456

**Published:** 2025-11-10

**Authors:** Ural Yunusbaev, Gabriela Romero Campos, Rajendran Sathishraj, Evgenii Liakh, John W. Raupp, Aleksey V. Zimin, Alina Akhunova, Dal‐Hoe Koo, Eduard Akhunov

**Affiliations:** ^1^ Department of Plant Pathology Kansas State University Manhattan Kansas USA; ^2^ Wheat Genetics Resource Center Kansas State University Manhattan Kansas USA; ^3^ Department of Biomedical Engineering Johns Hopkins University Baltimore Maryland USA; ^4^ Integrated Genomics Facility Kansas State University Manhattan Kansas USA

**Keywords:** *Aegilops geniculata*, crop wild relative, genome assembly, homoeolog expression bias, homoeologous chromosomes, population genomics, structural rearrangements, wheat genetic resources

## Abstract

*Aegilops geniculata* Roth is a tetraploid (M^g^M^g^U^g^U^g^; 2*n* = 4*x* = 28) wild relative of wheat and a valuable source of genetic diversity for improving agronomic traits. We present a high‐quality homoeolog‐resolved assembly and annotation of the *Ae. geniculata* genome and use it to study the function, origin and evolution of the M^g^ and U^g^ genomes. Comparative genomics revealed that the U^g^ genome has undergone extensive structural rearrangements (SRAs), which were inherited from its diploid ancestor. Chromosomes 4U^g^ experienced the most extensive SRAs, including translocations from chromosomes 1, 2, 6 and 7, as well as a pericentric inversion that repositioned the centromere closer to the chromosome terminus. The M^g^ genome had two large‐scale translocations, which likely occurred after polyploidization or in its immediate diploid ancestor. These SRAs resulted in the redistribution of genes among the homoeologous chromosomes, especially affecting the disease resistance genes. Although SRAs altered the expression and H3K4me3 marks of homoeologous genes relative to non‐rearranged regions, the overall balance of homoeolog expression and active chromatin remained stable, suggesting selective pressure to maintain gene dosage balance. Population genomic analyses of *Ae. geniculata* and its diploid ancestors, *Ae. comosa* (MM) and *Ae. umbellulata* (UU), suggest that *Ae. geniculata* originated in Western Anatolia. The genomic resources developed in this study will accelerate trait discovery, gene mapping and the transfer of beneficial alleles from this wild relative into wheat.

## 1 Introduction

*Aegilops geniculata* Roth (M^g^M^g^U^g^U^g^; 2*n* = 4*x* = 28) is an annual self‐pollinating natural allotetraploid derived from hybridization between *Ae. umbellulata* (UU; 2*n* = 2*x* = 14) and *Ae. comosa* (MM; 2*n* = 2*x* = 14) (Kihara 1954; Kimber et al. 1988). Previous phylogeographic analyses on the basis of AFLP/cDNA markers placed the *Ae. geniculata* origin to the eastern Mediterranean region (Arrigo et al. 2010) and revealed two highly differentiated sub‐populations south and north of the Bosphorus Strait distributed around the Mediterranean region (Van Slageren 1994). Extensive variation in morphological and physiological traits has been uncovered in populations sampled across the species geographic range suggestive of diverse mechanisms driving local adaptation (Zaharieva, Gaulin, et al. 2001; Bandou et al. 2009). *Ae. geniculata* is considered as a state noxious weed in California and Oregon because of its ability to hybridise with wheat (APHIS 2017). Broad adaptive potential and resistance to multiple agricultural pathogens make *Ae. geniculata* also a source of novel diversity for wheat improvement. Development of genomic resources for this grass, therefore, will be valuable for effective preservation of its diversity, monitoring its dispersal to non‐native habitats, understanding evolutionary processes driving its spread and adaptation, and effective usage of its diversity for breeding.

On the basis of crossability with wheat, *Ae. geniculata* belongs to tertiary gene pool and is considered a valuable source of novel, agronomically beneficial alleles for disease and pest resistance (Gill et al. 1985; Zaharieva, Monneveux, et al. 2001; Tiwari et al. 2015), tolerance to drought, heat, salt, and cold stress (Zaharieva, Gaulin, et al. 2001; Pradhan et al. 2012a, 2012b) and unique source of a gene capable of promoting recombination between homoeologous chromosomes (Koo et al. 2017; Gill et al. 2021). To facilitate introgression of genes from this wild relative, Friebe et al. (1999) developed a complete set of wheat‐*Ae. geniculata* disomic addition lines, several ditelosomic additions and disomic substitution lines. A number of disease resistance genes were introgressed into bread wheat from *Ae. geniculata* over the years. The stripe rust resistance genes *Yr40 and Lr57* (Kuraparthy et al. 2007) and stem rust resistance gene *Sr53* (Liu et al. 2011) were derived from the 5M^g^ chromosome. The *Pm29* gene conferring resistance to powdery mildew was introgressed from chromosome 6U^g^ (Zeller et al. 2002; Stoilova and Spetsov 2006). Chromosome 7M^g^ of *Ae. geniculata* was incorporated as a disomic addition and substitution line into common wheat, which not only enhanced wheat resistance to powdery mildew and Fusarium Head Blight, but also increased the grain weight (Wang et al. 2016, 2020, 2021; Yang et al. 2022). *The* 3M^g^ addition line conferred resistance to stripe rust and powdery mildew in bread wheat (Wang et al. 2021). Introgression of 2U^g^, 4U^g^, 5U^g^, 7U^g^, 2M^g^ and 7M^g^ chromosomes from *Ae*. *geniculata* increased the seed protein content of bread wheat (Rakszegi et al. 2017). Notably, the chromosome 5M^g^ of *Ae. geniculata* offers a valuable yet not characterised Homoeologous Pairing Promoter gene/s (*HPP‐5M* ^*g*^) that induces homoeologous recombination even in the presence of *Ph1* gene in wheat (Koo et al. 2017, 2020; Gill et al. 2021). A genetic system was developed utilising the *HPP‐5M* ^*g*^ for promoting homoeologous recombination and gene transfer in wheat (Koo et al. 2020; Gill et al. 2021).

The effective utilisation of wild relatives' genetic diversity in breeding requires the development of high‐quality genome assemblies and accurate annotation of their functional features. Recent advances in long‐ and short‐read sequencing technologies, along with the development of bioinformatical tools, provide an opportunity for assembling even the most complex, highly repetitive grass genomes at relatively low cost. A number of large complex genomes from *Triticeae* (Li et al. 2022; Grewal et al. 2024; Liu et al. 2024), including the two diploid ancestors of the M and U genomes *Ae. comosa* (Li, Rehman, et al. 2024) and *Ae. umbellulata* (Singh et al. 2024), respectively, have been assembled in recent years using the combination of PacBio HiFi reads followed by ordering contigs along chromosomes using Hi‐C data. The quality and contiguity of genomes assembled using this combination of technologies provide accurate ordering of nearly all genes along chromosomes and phasing information for contigs from different homoeologous chromosomes. The developed genome assemblies open doors to genome‐scale analyses of genetic diversity using cost‐effective low‐pass or reduced‐complexity genome sequencing to support characterisation of existing genebank collections, study evolutionary dynamic of wild populations in the context of crop domestication and improvement (Russell et al. 2016; He et al. 2019; Zhou et al. 2020; Zhao et al. 2023; Cavalet‐Giorsa et al. 2024), prioritise germplasm for breeding (Adhikari et al. 2022; Bohra et al. 2022; Wang, Bernardo, et al. 2023; Nyine et al. 2025) and conservation (Mascher et al. 2019; Khoury et al. 2022). The chromosome‐scale assemblies are an indispensable tool for tracing genome‐scale introgression events at the early stages of germplasm development to ensure effective transfer of wild relative diversity into adapted germplasm (Grewal et al. 2020; Nyine et al. 2020), mapping introgressed regions associated with beneficial traits (Nyine et al. 2021, 2025), and developing markers to tag beneficial introgressions or shorten introgressions to reduce linkage drag.

Here, we used a combination of Hi‐Fi reads with Hi‐C proximity ligation sequence data to generate a high‐quality genome assembly of the allotetraploid *Ae. geniculata* accession TA2899. This accession was used for mapping the Homoeologous Pairing Promoter gene/s (*HPP‐5M* ^*g*^) and developing new *HPP‐5M* ^*g*^ genetic stocks supporting effective recombination with wild ancestors of wheat (Koo et al. 2020; Gill et al. 2021). The multi‐tissue RNA sequencing using short‐read and Iso‐Seq approaches, along with the *Triticeae*‐specific protein datasets, were used for evidence‐based annotation of the genome. The ChIP‐Seq approach with CENH3 antibodies was used to identify centromeres in *Ae. geniculata*, permitting accurate chromosome assignment of structurally rearranged chromosomes in the U^g^ genome. Comparative genomics with wheat and the diploid ancestors of the M and U genomes showed that the majority of structural re‐arrangements (SRAs) between the M^g^ and U^g^ genomes occurred in the diploid ancestors of the U genome, *Ae. umbellulata*, with only a limited number of rearrangements potentially associated with post‐polyploidization events in the M^g^ genome. The analyses of the genomic distribution of genes showed a strong impact of structural rearrangements on chromosomal gene content, especially reflected in the redistribution of disease resistance genes between the M^g^ and U^g^ genomes. The distribution of the H3K4me3 epigenetic marks associated with actively expressed genes and transcriptome analyses demonstrated that structural rearrangements differentiating the U^g^ and M^g^ genomes have an impact on the transcriptional activity of duplicated homoeologous genes. The analyses of population‐level genetic diversity in *Ae. geniculata* identified a group of accessions that likely represented founders of extant *Ae. geniculata* accessions. These founders show broad geographic distribution, evidence of admixture with local populations and carry haplotypes in both M^g^ and U^g^ genomes that are most closely related to the accessions of *Ae. umbellulata* and *Ae. comosa* from Anatolia. The developed reference genome provides a powerful tool for accelerated trait discovery and characterisation in *Ae. geniculata* and the introduction of new diversity into wheat improvement programs.

## 2 Results

### 2.1 *Aegilops geniculata* Genome Assembly

To assemble the tetraploid genome of *Ae. geniculata* (accession TA2899), we generated ~296 Gb HiFi read data (Table S1), providing ~30× genome coverage. Using Hifiasm v. 0.19.5 (Cheng et al. 2021) with default settings, we have produced 1442 contigs with a mean contig length of 5.6 Mb and N50 of 14.8 Mb. The total length of assembled contigs was 8.1 Gb (Table S1). The contigs and Hi‐C data (~445 Gb) were used for scaffolding with HiRise software (Putnam et al. 2016), resulting in 14 chromosome‐scale pseudomolecules (7 per subgenome) and 453 unplaced contigs (Table S1 and Figure S1). Adding those unplaced contigs totals only ~41 Mb (Table S1), indicating that > 99% of the genome is anchored.

The pseudomolecules were assigned to chromosomes using skim‐seq data (0.01× genome coverage) generated for 13 disomic addition (DA) lines of *Ae. geniculata* (accessions TA2899) in the background of cultivar ‘Chinese Spring’ (Friebe et al. 1999). No DA line was available for the 6U^g^ chromosome of *Ae. geniculata*. The skim‐seq reads were mapped to the RefSeq v.2.1 combined with 14 pseudomolecules of *Ae. geniculata* and the mean depth of read coverage was calculated in 50 Mb non‐overlapping windows. The analyses of read depth coverage were used to assign pseudomolecules to the M^g^ and U^g^ genome chromosomes (Figure S2).

**FIGURE 1:**
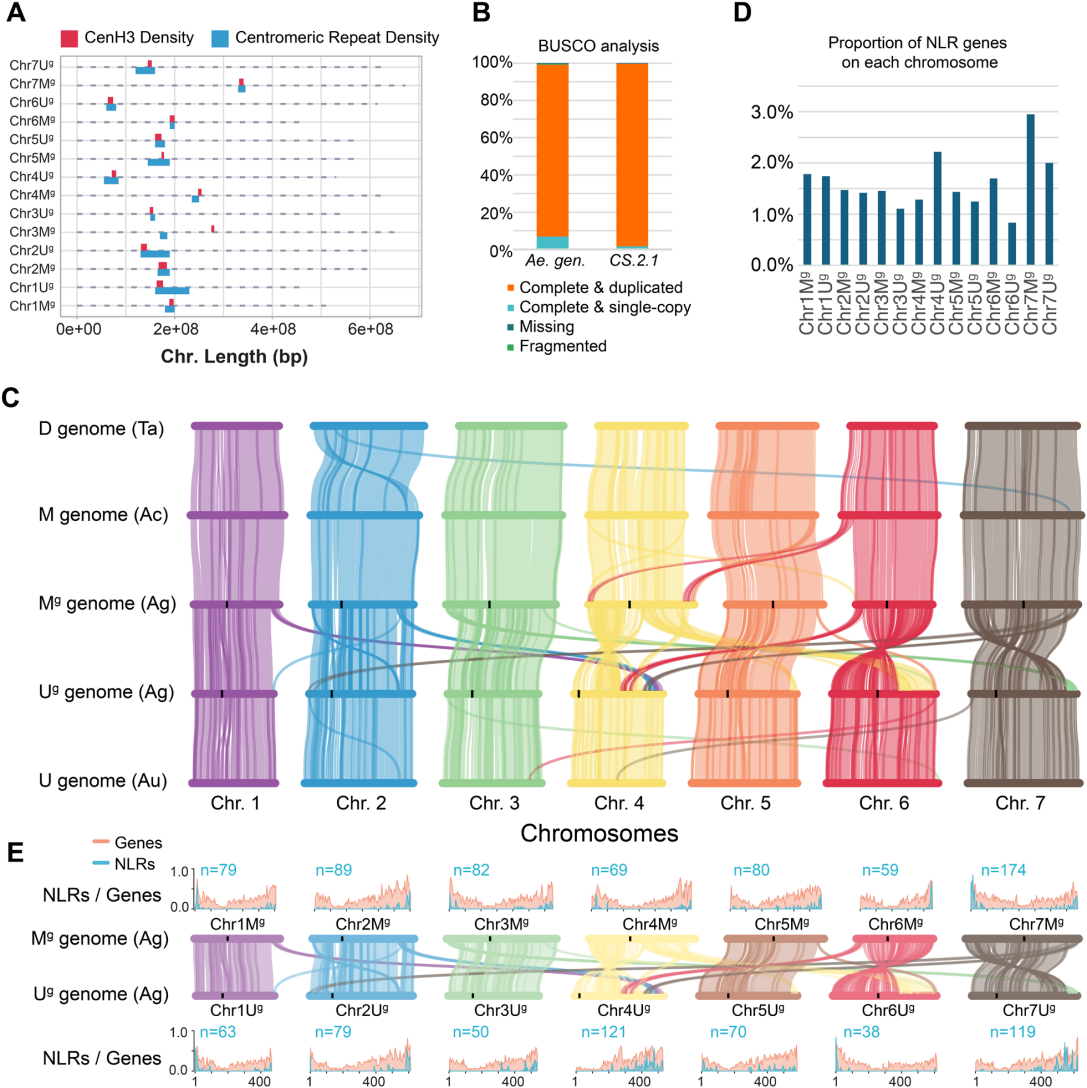
Annotation and comparative genomics of *Ae. geniculata* genome. (A) Location of centromeric repeats relative to the CENH3 ChIP‐seq peaks. In chromosome 3M^g^, the CENH3 peak is shifted relative to the location of centromeric TEs. (B) BUSCO summary statistics for the *Ae. geniculata* genome assembly in comparison with the completeness of *T. aestivum* genome assembly (CS v.2.1). (C) Synteny among the M^g^ and U^g^ genomes of *Ae. geniculata*, U genome of *Ae. umbellulata* (Au), M genome of *Ae. comosa* (Ac) and D genome of *T. aestivum* (Ta) (CS v. 2.1). The CENH3 peak locations are shown by black bars. (D) Proportion of disease resistance genes from the NLR family on each chromosome relative to the total number of predicted genes. (E) The density distribution for NLRs and predicted genes in the M^g^ and U^g^ genomes of *Ae. geniculata*. The number of predicted NLR gene models is shown for each chromosome.

The functional centromeres in *Ae. geniculata* were identified using ChIP‐seq with antibodies developed against the wheat CENH3 (Nagaki et al. 2003; Koo et al. 2015). Two replicates of CENH3 ChIP‐seq data were aligned to the *Ae. geniculata* genome. The depth of read coverage in 1 Mb windows was used to identify the centromere midpoints (Walkowiak et al. 2020). Both replicates revealed consistent peak positions across chromosomes (Figures 1A, S3, and S4, Table S2). Each chromosome had a single peak, except for chromosome 5M^g^, which had two closely spaced peaks. Except for chromosome 3M^g^, the CENH3 peaks were enriched for Cereba and Quinta transposable elements (Table S2, Figures S3 and S4) (Li et al. 2013). On chromosome 3M^g^, however, the CENH3 and centromeric transposon peaks were shifted, indicating a recent centromere repositioning (Figure 1A) that provided limited time for TE accumulation in the CENH3‐bound region.

### 2.2 *Ae. geniculata* Genome Annotation

The evidence‐based genome annotation was performed with EviAnn (Zimin et al. 2025), using a *Triticeae*‐specific (*Aegilops*, *Dasypyrum*, *Thinopyrum* and *Triticum*) set of 913 360 protein sequences from the NCBI database, along with the RNA‐seq data generated for multiple tissues of *Ae. geniculata* (Tables S3 and S4). RNA‐seq data were obtained from seven distinct tissues sampled across three developmental stages: leaf and root at the seedling stage; flag leaf, flag sheath and pre‐anthesis spikes at the pre‐anthesis stage; and whole spike and stem at the heading stage (Table S3). A total of 1 067 829 413 paired‐end 2 × 150 bp RNA‐seq reads were aligned to the *Ae. geniculata* genome (Dobin et al. 2013). In addition, 18 Gb of long‐read Iso‐seq data was generated using PacBio for RNA extracted from pre‐anthesis spikes and seedling roots (Table S4). Obtained 5 257 083 HiFi reads were Poly(A)/primers trimmed and clustered before alignment into 224 346 high‐quality transcripts (Table S4) using the PacBio SMRT Link software v11.0.

In total, we have identified 72 091 protein‐coding genes, with 36 420 genes from the M^g^ genome and 35 276 genes from the U^g^ genome, and 395 genes from the contigs not assigned to the chromosomes (Table S5). The BUSCO analyses showed that 99.2% of genes are complete and that 92.3% of annotated genes are duplicated, consistent with the presence of M^g^ and U^g^ genome homoeologous copies of genes (Figure 1B and Table S6). Only a small fraction of BUSCO genes (0.8%) was classified as fragmented or missing, indicative of the high quality of *Ae. geniculata* genome assembly and annotation. To test whether the genes present in only one of the two genomes are indeed single‐copy genes, we compared the read coverage depth and number of variants between two groups of genes—(1) single‐copy genes and (2) all other genes in the genome—using alignments generated by mapping HiFi reads back to the *Ae. geniculata* assembly. If single‐copy genes resulted from the collapse of duplicated homeologs into a single assembly, we would expect increased coverage depth or a higher number of variants in single‐copy genes compared to other genes. However, we did not detect significant differences in coverage (13.1 vs. 13.0, *p* = 0.11) or number of SNPs (0.05 SNPs/gene vs. 0.02 SNPs/gene, *p* = 0.18) between these two groups, supporting the validity of the single‐copy gene models.

Annotation of transposable elements (TEs) was performed using RepeatMasker (Table S7). Although total proportions of sequence represented by TEs in the M^g^ (51%) and U^g^ (49%) genomes were similar, we found that most classes of TEs have undergone significant genome‐specific changes in copy number (Table S7), with the most striking differences observed for Class I TEs. There was a significant increase (17.5%) of *Gypsy* LTRs (RLG) in the U^g^ genome relative to M^g^ and an increase (38.8%) of *Copia* LTRs (RLC) in the M^g^ genome relative to U^g^. Some of these increases contribute to changes in chromosome sizes observed between the U^g^ and M^g^ genomes (Table S8).

### 2.3 Structural Rearrangements in the *Ae. geniculata* Genome

To explore the evolution of genome structure in *Ae. geniculata*, we performed comparative synteny analyses between the M^g^ and U^g^ genomes. Using reciprocal best BLASTN hit analysis (Figure S5), we have identified 21 167 syntenic genes (Table S9). A total of 13 large‐scale SRAs were detected among the homoeologous chromosomes from the M^g^ and U^g^ genomes (Table S10, Figures S6 and S7). The SRAs were detected for all homoeologous chromosome pairs, with most rearrangements located on chromosomes from groups 4 and 6 (Figure 1C). The 4 U^g^ chromosome is composed of segments showing synteny with chromosomes 1M^g^, 2M^g^, 6M^g^ and 7M^g^. The 4M^g^‐4U^g^ chromosome synteny analysis points at a pericentric inversion on 4 U^g^ that resulted in the re‐positioning of the centromere from the middle portion of the chromosome to the chromosome terminus (Figure 1C). In addition, 4M^g^‐4U^g^ and 4M^g^‐6U^g^ comparisons showed translocation of a segment from chromosome 4 to 6 in the U^g^ genome. The 3M^g^‐7U^g^ comparison showed translocation of a short arm segment from chromosome 3 to the distal long arm of chromosome 7 in the U^g^ genome. In addition, intrachromosomal translocation with 7U^g^ involving translocation from the short arm of 7U^g^ to the long arm of the same chromosome was detected.

Comparison of *Ae. geniculata* gene synteny was performed using the previously published diploid genomes of *Ae. comosa* (M genome) (Li, Rehman, et al. 2024) and *Ae. umbellulata* (U genome) (Singh et al. 2024) that share homologous genomes with *Ae. geniculata*. In addition, syntenic analyses included the D genome of Chinese Spring reference (Zhu et al. 2021), as the only one of three wheat genomes that preserved synteny with its diploid ancestor, *Ae. tauschii* (Luo et al. 2017). These analyses indicate that nearly all SRAs observed between the M^g^ and U^g^ genomes occurred in the diploid ancestor represented in our analyses by *Ae. umbellulata* (Singh et al. 2024). The SRAs differentiating the U^g^ genome from the M^g^ genome are also clearly detectable in comparisons of the U^g^ genome with the wheat D genome (Figure 1C). No post‐polyploidization structural re‐arrangements that are unique to the U^g^ genome in *Ae. geniculata* were detected.

The M^g^ genome of *Ae. geniculata* and the ancestor represented by *Ae. comosa* (Li, Rehman, et al. 2024) was mostly collinear, except for three structural rearrangements unique to the M^g^ genome of *Ae. geniculata* (Table S10). The translocation of two terminal segments from the short arm of chromosome 6M^g^ to the ends of chromosome 4M^g^, and duplication of a terminal segment on the long arm of chromosome 5M^g^ to chromosome 4M^g^ appears to have happened after polyploidization or in the direct ancestor of the *Ae. geniculata* M^g^ genome, if it was different from the sequenced accession of *Ae. comosa* (Figure 1C).

We tested whether the SRAs in the *Ae. geniculata* genome had any impact on the distribution of genes among chromosomes, focusing on disease resistance genes from the NBS‐LRR (NLR) family of immune receptors that are abundant at the distal regions of chromosomes (Luo et al. 2013; Steuernagel et al. 2020; Li et al. 2022). Terminal translocations from chromosomes 1U^g^, 2U^g^, 6U^g^ and 7U^g^ to chromosome 4U^g^, and from 3U^g^ to 7U^g^, changed the content and distribution of NLR genes. The 1M^g^‐1U^g^, 2M^g^‐2U^g^ and 5M^g^‐5U^g^ chromosome pairs, which experienced SRAs of a smaller scale compared to other chromosomes, had about equal proportions of genes represented by NLRs, 1.8%, 1.4%, and 1.3%, respectively (Figure 1D,E, Table S11). However, the relative proportion of NLRs between the remaining pairs of homoeologous chromosomes was quite distinct. In chromosome 4U^g^, the proportion of NLR genes increased from 1.3% to 2.2% relative to 4M^g^, and the distribution of NLR genes shifted from the 4U^g^ short arm to the 4 U^g^ long arm (Figure 1D,E and Table S11). Likewise, NLR gene proportions reduced from 1.7% on 6U^g^ to 0.8% on 6M^g^, and from 2.9% on 7M^g^ to 2.0% on 7U^g^.

### 2.4 Anchoring Candidate Gene Intervals to the *Ae. geniculata* Genome

*Ae. geniculata* has been extensively used as a source of novel disease resistance genes (R). The availability of a chromosome‐level reference genome assembly for *Ae. geniculata* enables more precise physical anchoring of resistance genes, eliminating dependence on proxy genomes. We used *Ae. geniculata* genome assembly to identify genomic intervals harbouring R genes previously mapped using molecular markers (Table S12). The leaf and stripe rust resistance genes *Lr57* and *Yr40*, both derived from chromosome 5M^g^ were initially mapped to the distal short arm of 5M^g^S (Kuraparthy et al. 2007; Steadham et al. 2021). In the *Ae. geniculata* genome, these two genes mapped to a 6.5–9.6 Mb interval, harbouring three tentative R gene candidates. The *Lr76* and *Yr70* genes were mapped to the short arm of chromosome 5U in *Ae. umbellulata* using markers derived from wheat chromosome 5D (Bansal et al. 2020). In 5U^g^, these loci mapped to the 13.2–26.7 Mb interval, including 14 candidate R genes. The *Sr53* resistance gene that was mapped to the proximal region of 5M^g^L using sequence‐tagged site and simple sequence repeat markers (Liu et al. 2011; Tiwari et al. 2015) was anchored to the 246.8–383.4 Mb interval on 5M^g^ harboring 38 candidate R genes.

Additionally, the *Ae. geniculata* reference genome facilitated cross‐validation of gene positions across sub‐genomes. For example, the leaf rust resistance gene *Lr9*, introgressed from *Ae. umbellulata* chromosome 6U into wheat chromosome 6B and recently cloned (Wang, Abrouk, et al. 2023), was mapped to chromosome 4U^g^ in the *Ae. geniculata* genome assembly. This discrepancy is likely caused by mis‐identification of chromosome 4U as 6U in the prior assembly of the *Ae. umbellulata* genome (Abrouk et al. 2023; Singh et al. 2024). Also, it suggests that the presence of a genomic segment in the structurally re‐arranged 4U chromosome that has synteny with the group 6 chromosomes facilitated the transfer of *Lr9* into wheat. These analyses show that the *Ae. geniculata* reference genome provides a robust resource for gene mapping.

### 2.5 Gene Expression Dynamic Across Tissues in *Aegilops geniculata*

We utilised RNA‐seq data from seven tissues to investigate tissue‐specific expression dynamics of duplicated homoeologous genes (HG) and genome‐specific unique genes (UG) from the U^g^ and M^g^ genomes. The HG and UG sets were defined on the basis of reciprocal best‐BLASTN hits. A total of 1.07 billion Illumina paired‐end reads (2 × 150 bp) were used for the quantification of gene expression by aligning to the set of 70 231 transcripts (Table S13). After filtering transcripts for cumulative expression of at least 10 TPM across all tissues, 36 070 (51.36%) were retained for further analyses. In total, expression values were obtained for 26 424 HGs, 4945 genes unique to the M genome and 4645 unique to the U genome.

The gene expression varied across the tissues, with some genes being expressed in all tissues, whereas others are tissue‐specific (Table S13). The total number of expressed genes was similar across the different tissues, with 70.6% of them being expressed in all tissues. Only 3.3% of the genes were exclusively expressed in a single tissue. Among seven tissues, seedling stage root and whole spike stand out, with 50.3% (590 genes) and 35.8% (420 genes) of all tissue‐specific genes expressed in these two tissues (Table S14).

**FIGURE 2:**
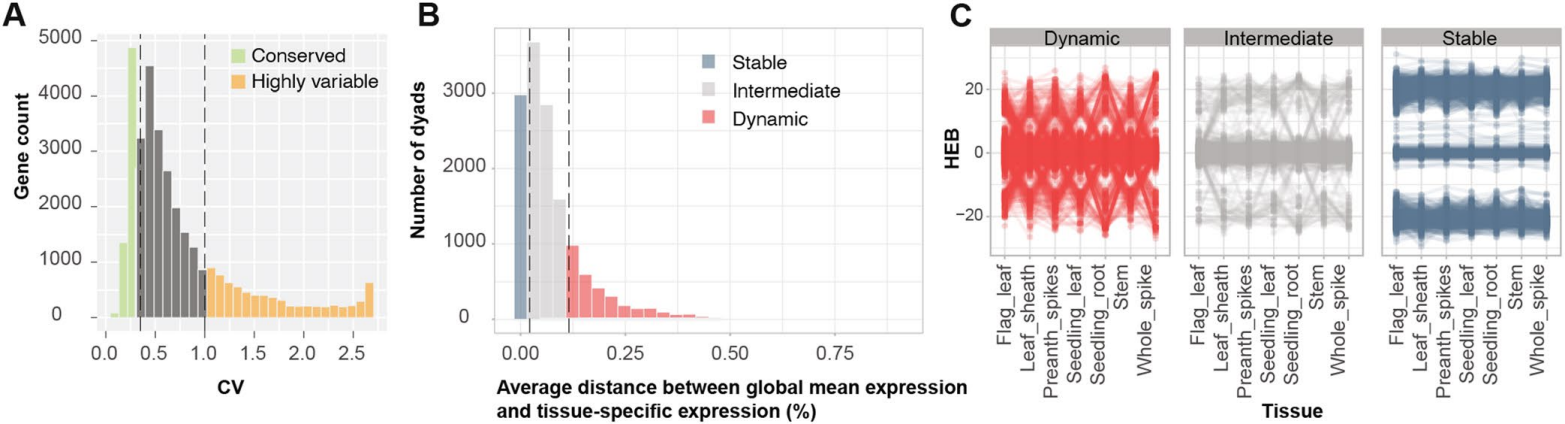
Gene expression dynamics in *Ae. geniculata*. (A) Distribution of the coefficient of variation (CV) for gene expression values across tissues. CV was used to classify genes into conserved and highly variable groups. (B) Classification of dyads into three categories (stable, intermediate and dynamic) on the basis of the consistency of homoeologous expression bias across tissues, which was performed on the basis of the average Euclidean distances between global and tissue‐specific normalised expression values. (C) Homoeolog expression bias (HEB) across tissues for the representative sets of dyads selected from stable, intermediate and dynamic categories of dyads defined in Figure 2B.

On the basis of variability of expression across tissues assessed by calculating the coefficient of expression variation (CV), genes were classified into three categories: highly variable (CV > 1), intermediate (0.35 < CV < 1) and conserved (CV < 0.35) (Figure 2A and Table S15). This classification separates genes with a high CV, mostly associated with tissue‐specific characteristics or specific stages of plant development, from genes with low CV, which tend to play an essential role in all tissues. Out of 36 070 genes, 21.1% exhibited highly variable patterns of expression across tissues, whereas 24.9% showed conserved expression (Table S15). Further, we compared expression CV in the homoeologous gene pairs (henceforth, dyads) from the M^g^ and U^g^ genomes. Among the 21 167 dyads, both homoeologous copies were not expressed in 7005 (33.1%) dyads and excluded from further analyses (Table S16). Of the 12 262 dyads with at least one gene copy expressed, a total of 817 (6.6%) homoeologs in the M^g^ genome and 1083 (8.8%) homoeologs in the U^g^ genome were not expressed or expressed at a low level. Of 12 262 dyads, 9836 (80.2%) have gene pairs that were classified within the same expression CV category. Only 11 dyads had gene pairs showing a strong contrast in expression CV, where one homoeologous copy was classified as highly variable, and another one was classified as conserved (Table S16).

The relative expression of genes in dyads across tissues was evaluated by calculating the homoeolog expression bias (HEB), where HEB = 0 corresponds to the lack of biased expression, and the positive and negative HEB values correspond to M^g^—and U^g^‐genome biased expression, respectively (Figure S8). On average, 11 074 genes across tissues showed balanced expression, and 1739 and 1348 genes showed M^g^‐ and U^g^‐dominant expression, respectively (Table S17). Though the proportions of dyads showing balanced, M^g^‐ or U^g^‐dominant expression were not substantially different among tissues, the assignments of some dyads into one of the three HEB categories changed from tissue to tissue.

To investigate the HEB patterns across tissues, we classified the dyads into stable, dynamic or intermediate classes using the previously described approach (Ramírez‐González et al. 2018), which was modified for handling homoeologous genes from a tetraploid genome. The classification was based on the metric that assesses the deviation of each dyad's gene expression in individual tissues from the global mean, where stable genes show expression closer to the global mean whereas dynamic genes show the strongest deviation from the global mean. We have identified 2833 dyads with dynamic expression variation, 8355 dyads with intermediate expression variation, 2974 dyads with stable expression variation across tissues (Figure 2B,C).

### 2.6 Impact of SRAs on Transcriptionally Active Chromatin and Gene Expression in *Aegilops geniculata*

Previous studies have suggested that large‐scale SRAs can influence chromatin states, potentially leading to changes in gene expression (Alonge et al. 2020; Li, Wang, et al. 2024). We used RNA‐seq and H3K4me3 ChIP‐seq data to investigate the impact of SRAs between the M^g^ and U^g^ genomes on gene expression and transcriptionally active chromatin. H3K4me3 peaks usually coincide with actively expressed genes and span up to 1 kb regions downstream of the transcription initiation sites (Zhang et al. 2009). ChIP‐seq data were used to detect the presence/absence of peaks within 500 bp regions around the start of coding regions. To compare transcriptionally active chromatin between the homoeologous pairs of genes, we calculated the log2‐ratio of area covered by the ChIP‐seq reads in the M^g^ and U^g^ genome homoeologs, referred to as the H3K4me3 Peak Homoeologous Bias (HPHB) (for details, see Methods).

Comparison of various metrics calculated using the gene expression and H3K4me3 data for the two groups of genes or dyads, one from the structurally rearranged and another from the non‐rearranged regions, showed statistically significant differences in TPM (*p* = 1.29E−03), proportion of expressed genes (*p* = 4.10E−03), proportion of genes with H3K4me3 peaks (*p* = 1.00E−04), and proportions of M^g^‐ or U^g^‐dominant homoeologs (*p* = 3.45E−02) (Table 1). However, the relative levels of expression (HEB) and relative H3K4me3 peak area (HPHB) between the homoeologs in dyads did not show significant changes between structurally rearranged and non‐rearranged genomic regions.

**TABLE 1:** Comparison of gene expression and H3K4me3 epigenetic marks between the groups of genes or dyads located in the SRA and non‐rearranged genomic regions.

Gene expression or active chromatin metric	Genomic features	*N* genes in non‐rearranged regions	*N* genes in rearranged regions	*p* (adj)^a^	Significance
HEB	Dyads	11 519	7317	1.56E−01	ns
TPM	Genes	23 038	14 634	1.29E−03	**
HPHB	Dyads	11 519	7317	2.94E−01	ns
H3K4me3 summit	Genes	23 038	14 634	2.03E−10	**
Proportion of expressed genes	Genes	23 038	14 634	4.10E−03	**
DCB^b^	Genes	11 519	7317	3.45E−02	*
H3K4me3 status	Genes	23 038	14 634	1.00E−04	**

Abbreviation: ns, non‐significance.

^a^ Non‐parametric Kruskal–Wallis test was used for assessing significance.

^b^ DCB—Dominance Category Bias compares the distribution of dyads with dominant and biased expression between the SRA and non‐SRA regions.

* ≤ 0.05.

** ≤ 0.01.

**FIGURE 3:**
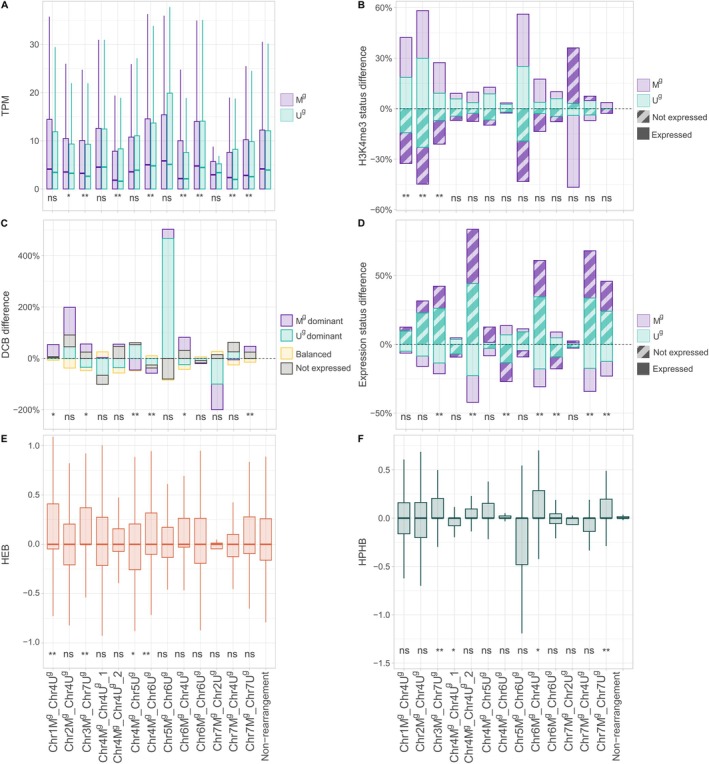
Gene expression and homoeologous bias across 13 SRA genomic regions compared to non‐rearranged regions. (A) Transcript abundance (TPM) in the M^g^ and U^g^ genomes. (B) Changes in the proportions of genes having the H3K4me3 peaks within 500 bp coding regions from the start codon. The proportions are shown as changes relative to non‐rearranged regions. The presence of H3K4me3 peaks is shown for both expressed and non‐expressed gene homeologs in the M and U genomes. (C) Changes in the proportions of homoeologous dyads in SRA regions classified as M^g^‐dominant, U^g^‐dominant or balanced relative to the same classes of dyads in the control group. The control includes dyads from unrearranged parts of the genome. (D) The change in the proportions of expressed or silenced genes in the structurally rearranged regions relative to the rest of the genome. (E) Homoeologous expression bias (HEB). (F) H3K4me3 peaks homoeologous bias (HPHB) is shown for 13 rearranged blocks compared to the non‐rearranged portion of the genome. Statistical significance was evaluated with the Wilcoxon signed‐rank test (FDR‐adjusted). *p < 0.05; **p < 0.01; ns = not significant.

Further, we have compared the same metrics between the non‐rearranged parts of the genome and each of the 13 SRA genomic regions (Figure 3). Most of the rearranged regions showed a significant shift in the distribution of TPM values relative to the TPM values of homoeologous genes located outside of the rearranged regions (Figure 3A). The direction of TPM changes was mostly towards reduced expression in the rearranged regions, with six out of eight regions with significant changes showing reduced TPM values compared to the non‐rearranged control (Figure 3A). In most regions, a significant increase was observed in the number of silenced genes (Figure 3D) or dyads that showed M^g^‐dominant expression (Figure 3C). Only three structurally rearranged regions showed significant changes in the presence/absence of H3K4me3 peaks at the start of the gene (Figure 3B), suggesting that expression changes might be associated with other factors.

Comparison of the HEB and HPHB metrics between the dyads from the structurally rearranged and non‐rearranged regions, however, showed no difference for most of the regions (Figure 3E,F), suggesting that changes in gene expression or H3K4me3 marks occurring in one of the homoeologs are likely compensated by comparable changes in another.

### 2.7 Population Genomics of *Ae. geniculata*

To investigate population structure, genetic diversity and evolutionary history of the M^g^ and U^g^ genomes in *Ae. geniculata* and its diploid ancestors, we used the sequence‐based genotyping data generated for *Aegilops* species (Adhikari et al. 2023) (Tables S18 and S19). Filtering SNPs for MAF ≥ 0.5 and missing genotype call rate less than 50% resulted in 20 055 and 46 986 SNPs in the M^g^ and U^g^ genomes, respectively. This SNP dataset was used in PCA to evaluate the samples for the correctness of assignment to species, specifically focusing on *Ae. geniculata* and the diploid ancestors of the M (*Ae. comosa*) and U (*Ae. umbellulata*) genomes (Figures S9 and S10). In addition, to confirm the ploidy level of the analysed samples, we assessed the depth of read coverage distribution across the M^g^ and U^g^ genomes of *Ae. geniculata*.

PCA (Figures S9 and S10) showed that all accessions labelled as *Ae. comosa*, the diploid M genome ancestor, form a single cluster, indicating the lack of mislabelled samples in the dataset. These analyses identified two samples of *Ae. geniculata* (TA1711 and TA2043) and one sample of *Ae. umbellulata* (TA11104) that clustered with other species and, therefore, excluded from further analyses (Table S18). There were also four samples labelled as *Ae. geniculata* (TA1879, TA10847, TA2221 and TA10002) and four samples labelled as *Ae. umbellulata* (TA1854, TA1848, TA2633 and TA11097) that fell outside of the main PCA clusters formed by each of these two species. However, these samples did not show clear overlap with the clusters formed by other species, and therefore were not considered for further investigation (Table S20). In addition, two accessions TA11085 and TA2231, which were originally labelled as *Ae. peregrina* and *Ae. triuncialis*, respectively, clustered closely with the *Ae. geniculata* accessions and were added to the dataset as *Ae. geniculata* samples for downstream analyses. The resulting set included 18 *Ae. comosa*, 142 *Ae. geniculata* and 56 *Ae. umbellulata* accessions (Table S21). The genotype calls generated for this set include 20 149 SNPs in the M^g^ genome and 20 078 SNPs in the U^g^ genome with MAF ≥ 0.05 and missing genotype calls less than 50% (Table S22).

#### 2.7.1 Population Structure of *Ae. geniculata*

**FIGURE 4:**
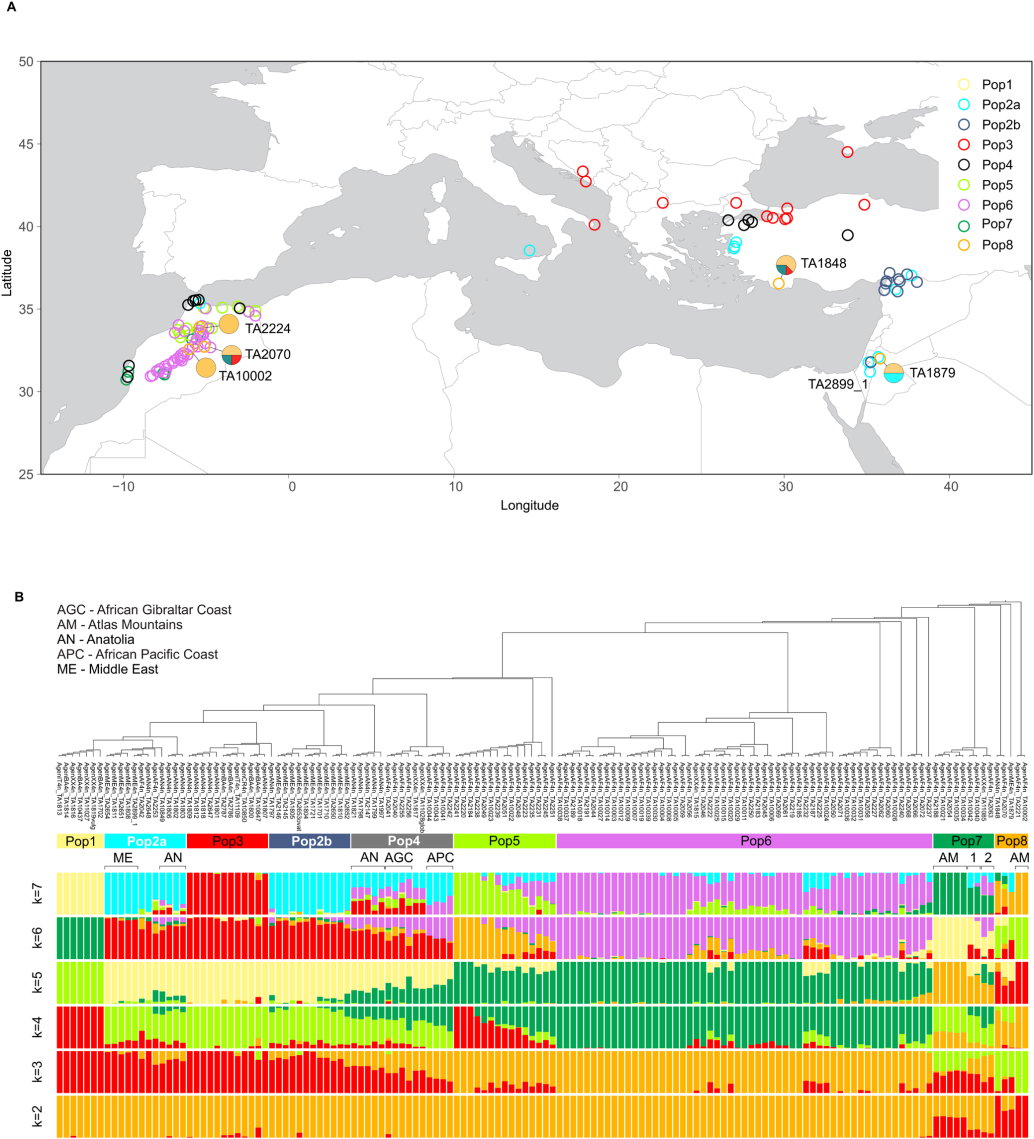
Population structure of 141 *Ae. geniculata* accessions. (A) Geographic distribution of 141 samples of *Ae. geniculata* and assignments of samples to 8 sub‐populations on the basis of the analyses of admixture and phylogenetic clustering presented in panel B. The accession numbers are provided for five accessions that showed an early split from the rest of the population, with most of their ancestry assigned to a separate cluster at *K* = 2. For each of these five accessions, the proportions of ancestry at *K* = 7 are shown as a pie chart. The colour‐coding of samples matches the colours used in panel B to designate eight sub‐populations. (B) Neighbour‐joining (NJ) tree was built using a distance matrix calculated on the basis of the Identity‐By‐State (IBS) allele counts. The estimation of the ancestry proportions in *K* populations for each accession that ranged from *K* = 2 to *K* = 7 was performed using Admixture. Accessions were split into eight subpopulations on the basis of Admixture and NJ tree clustering. The geographic origin of the subgroups of accessions within pop2a and pop4 is shown on the top left. The accessions from pop1 do not have information about the geographic origin and were obtained from various sources (Table S4).

Analysis of the population structure was performed using Admixture for values of *K* ranging from 2 to 9. The error on the basis of 5‐fold cross validation stabilised at *K* = 4 at 0.306 and started increasing at *K* = 8. The proportions of Q memberships for each sample in the *Ae. geniculata* population at values of K ranging from 2 to 7 were evaluated in relation to geography and phylogenic clustering, and all accessions were grouped into 8 subpopulations (Figure 4). At *K* = 2, five *Ae. geniculata* samples (pop8) from North Africa (TA10002, TA2221 and TA2070), western regions of Anatolia (TA1848) and the Middle East (TA1879) showed more than 63% of ancestry assigned to a separate cluster. Separation of these accessions from the remaining samples indicates their early differentiation and suggests that they likely represent an ancient ancestral population of *Ae. geniculata*. The broad geographic distribution of these accessions, ranging from Turkey (TA1848) and Jordan (TA1879) to Morocco (TA10002, TA2221 and TA2070), and the evidence of admixture with the local populations (Figure 4A) supports this hypothesis. In addition to these five accessions, nine accessions from pop7 sampled in North Africa (TA2063, TA11085, TA10040, TA10042, TA10034, TA10035, TA2054, TA10021 and TA2188) showed from 10% to 50% of their ancestry assigned to this cluster at *K* = 2. At *K* = 7, four out of nine accessions from pop7 showed mixed ancestry. Two samples from pop7‐1 (TA10040, TA10042) had 25.0% and 36.2% of their ancestry coming from pop2a and pop2b located in the Middle East. Two samples from pop7‐2 (TA2063, TA11085), in addition to showing 23.2% and 7.6% of their ancestry from pop2a/pop2b, had admixture with the closest North African population pop6.

At *K* = 7, we observed a clear separation of the European samples of *Ae. geniculata* from pop3 from the Middle Eastern accessions from pop2a/pop2b. Accessions located between the pop3 and pop2a/pop2b geographic regions had mixed ancestry from these populations and were included in pop4. Overall, the population pop4 included highly admixed accessions that all have parts of their ancestry assigned to pop6 in Africa and pop2a/pop2b in the Middle East. In addition, the pop4‐AN and pop4‐AGC groups had admixture from pop3 in Europe and pop5 in North Africa. We also detected two geographically close but genetically distinct populations in Morocco: low‐land population pop5 (mean elevation is 411 m above sea level) and high‐land population pop6 (mean elevation is 1073 m above sea level) (Figure 4B). These results might suggest the existence of two populations adapted to low and high‐land climatic conditions.

#### 2.7.2 Geographic Origin of *Ae. geniculata*

**FIGURE 5:**
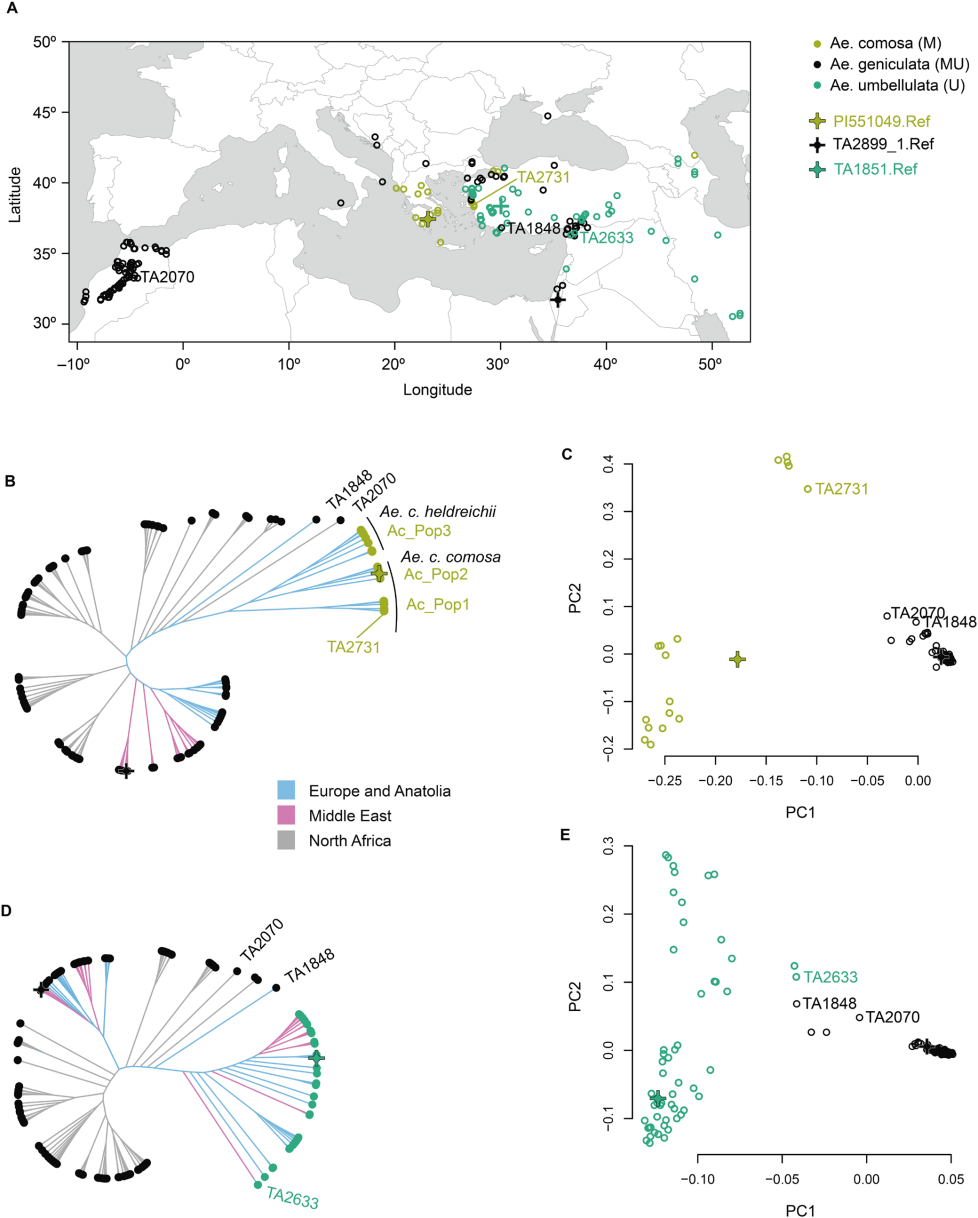
(A) Geographical distribution of the *Ae. comosa*, *Ae. geniculata*, and *Ae. umbellulata* accessions. (B) SNP‐based phylogeny of the *Ae. comosa* and *Ae. geniculata* accessions showing three subpopulations within the *Ae. comosa* as labelled on the tree. Accessions used to generate reference genome assemblies are indicated by crosses. The *Ae. comosa* accession TA2731 most closely related to *Ae. geniculata* belongs to subpopulation 1. The *Ae. geniculata* accessions TA1848 and TA2070 are the closest to the *Ae. comosa* branch. (C) SNP‐based PCA plot of the *Ae. comosa* and *Ae. geniculata* accessions. The plot shows three subpopulations within the *Ae. comosa* and the *Ae. comosa* TA2731 also drops closest to the *Ae. geniculata* cloud on the PC1 axis. (D) SNP‐based phylogeny of the *Ae. umbellulata* and *Ae. geniculata* accessions. The *Ae. umbellulata* accession TA2633 is the closest to *Ae. geniculata*. Notably, the *Ae. geniculata* accessions TA1848 and TA2070 are also located closest to the *Ae. umbellulata* branch. (E) SNP‐based PCA plot of the *Ae. umbellulata* and *Ae. geniculata* accessions. The plot shows the *Ae. umbellulata* TA2633 position closest to the *Ae. geniculata* cloud on the PC1 axis. The *Ae. geniculata* accessions TA1848 and TA2070 are closest to the *Ae. umbellulata* on the PC2 axis.

We further investigated the genetic diversity and phylogeny of *Ae. comosa*, *Ae. geniculata* and *Ae. umbellulata* accessions to identify the most likely geographic origin of *Ae. geniculata* (Figure 5). The SNP‐based phylogeny and PCA of the *Ae. comosa* and *Ae. geniculata* accessions identified three subpopulations within the *Ae. comosa*, as labelled on the tree (Figure 5B,C). The accession TA2731 from Anatolia, which clusters closest with *Ae. geniculata* on the PCA plot, belongs to subpopulation 1 (Ac_Pop1) of *Ae. comosa* ssp. *comosa*. The phylogenetic tree of *Ae. comosa* also supports the conclusion that Ac_Pop1, including four accessions TA2731, TA2732, TA2171, and TA2760, is the nearest to *Ae. geniculata* accessions. Geographically, Ae. comosa accessions TA2731, TA2732 and TA2171 are located in close proximity to each other on the western coast of Anatolia, whereas TA2760 is located in the Balkans across the Aegean Sea. Out of two *Ae. geniculata* accessions showing closest genetic distance to subpopulation 1 of *Ae. comosa*, TA1848 also originated in Anatolia, whereas TA2070 was sampled in North Africa. These two *Ae. geniculata* accessions are assigned to pop8 (Figure 4B), which showed clear genetic differentiation in the Admixture analyses at *K* = 2. The published genome of the accession PI551049 (Li, Rehman, et al. 2024) clustered with subpopulation 3 of the *Ae. comosa* ssp. *comosa* that is more distantly related to the *Ae. geniculata* M^g^ genome than TA1848 and TA2070. On the basis of these results, we conclude that subpopulation 1 of *Ae. comosa* is the most likely donor of the *Ae. geniculata* M^g^ genome.

SNP‐based phylogeny and PCA indicate that the *Ae. umbellulata* accession TA2633 from northern Syria is the closest relative of *Ae. geniculata* (Figure 5D,E). Among *Ae. geniculata* accessions, TA1848 from Anatolia and TA2070 from North Africa, are also positioned nearest to the *Ae. umbellulata* accessions on the phylogenetic tree. Notably, the M^g^ genomes of these *Ae. geniculata* accessions also showed the closest relatedness to *Ae. comosa*, the diploid ancestor of the M genome (Figure 5B,C). Together, these analyses of genetic diversity and population structure of *Ae. geniculata* and its diploid ancestors suggest *Ae. geniculata* originated in the eastern region of modern‐day Turkey.

## 3 Discussion

### 3.1 The Structure of Chromosomes From the M^g^ and U^g^ Genomes of *Ae. geniculata*

Here, we present a homoeolog‐resolved genome assembly and annotation of tetraploid *Ae. geniculata* generated using a combination of PacBio Hi‐Fi reads and Hi‐C scaffolding. The evidence‐based annotation of the genome was performed using the EviAnn pipeline (Zimin et al. 2025) provided high‐quality annotation of gene models, with 99.2% of annotated genes being complete. As expected for an allopolyploid genome, 92.3% of genes were identified as duplicated. Synteny analyses between the M^g^ and U^g^ genomes revealed extensive SRAs, particularly involving chromosomes 4U^g^ and 6U^g^. Because of these rearrangements, chromosome 4U^g^ consists of terminal gene‐rich segments of chromosomes translocated from 1U^g^, 2U^g^, 6U^g^ and 7U^g^. In addition, chromosome 4U^g^ has undergone a pericentric inversion, relocating a gene‐rich terminal region from the short arm to the middle part of the long arm. This inversion also resulted in centromere repositioning, as detected by ChIP‐seq with CENH3 antibodies, placing the centromere closer to the chromosome end. Recent analyses of the genetic map developed for the related tetraploid grass *Ae. biuncialis* (Lampar et al. 2025), which also carries the U^b^ and M^b^ genomes, showed that its M^b^ genome has nearly complete collinearity with the wheat D genome, consistent with the results obtained for *Ae. geniculata*. Except for a swapped identification of chromosomes 4U^b^ and 6U^b^, the U^b^ genome of *Ae. biuncialis* carried the same structural rearrangements detected in *Ae. geniculata*. The misidentification of chromosomes 4U and 6U in *Ae. biuncialis* (Lampar et al. 2025) and the previously sequenced *Ae. umbellulata* (Abrouk et al. 2023; Singh et al. 2024) is likely associated with a large‐scale SRA involving reciprocal translocations between these two chromosomes. By experimentally mapping centromeres using CENH3 ChIP‐seq, our study resolves this ambiguity.

The comparative genome analyses with the diploid *Ae. umbellulata* (U genome) (Singh et al. 2024) showed that all structural rearrangements detected in the U^g^ genome of *Ae. geniculata* occurred in the diploid ancestor. Combined with the results of Ae. *biuncialis* genetic map analyses (Lampar et al. 2025), this result suggests that the U genomes of both Ae. *biuncialis* and *Ae. geniculata* are contributed by the ancestral *Ae. umbellulata* population(s) that had similar chromosome structure, indicating that the SRAs detected in the U genome are likely fixed in *Ae. umbellulata*. Comparison with the diploid *Ae. comosa* (M genome) (Li, Rehman, et al. 2024) genome showed good collinearity with the M^g^ genome of *Ae. geniculata*, except for a few structural rearrangements that might potentially have happened after polyploidization and involved translocations from chromosomes 6M^g^ and 5M^g^ to chromosome 4M^g^. However, we cannot rule out the possibility that the diploid ancestor that contributed to the *Ae. geniculata* M^g^ genome also had similar rearrangements because the genotype of the sequenced *Ae. comosa* accession is not the closest ancestor of the *Ae. geniculata* M^g^ genome on the basis of the analyses of genetic diversity.

One of the consequences of SRAs in the *Ae. geniculata* genome is the alteration of synteny and gene distribution at the terminal regions of chromosomes involved in translocations. This effect is particularly evident for disease resistance genes from the NBS‐LRR family, which are highly enriched at the distal ends of large *Triticeae* chromosomes (Luo et al. 2013; Steuernagel et al. 2020; Li et al. 2022). These findings may have important practical implications for pre‐breeding efforts that use *Ae. geniculata* as a source of beneficial alleles for wheat improvement. Because of SRAs, genes located in regions of the U^g^ genome that have lost synteny with wheat chromosomes may become inaccessible for introgression into wheat. Consistent with this prediction, in crosses between wheat and Ae. biuncialis, wheat chromosomes paired more frequently with the Mb genome chromosomes than with the Ub genome chromosomes (Türkösi et al. 2022). Therefore, we might expect that the transfer into wheat of beneficial genes located in the SRAs of the Ug genome in Ae. geniculata and its diploid ancestor Ae. umbellulata may be challenging using conventional chromosome engineering approaches relying on the wheat ph1b mutant (Qi et al. 2007). However, partial chromosome synteny may be sufficient to reduce the impact of SRAs on introgression efficiency. The successful transfer of the *Lr9* gene (Wang, Abrouk, et al. 2023), which maps to the highly rearranged chromosome 4U^g^ of *Ae. geniculata* and was introgressed into wheat chromosome 6B, is likely due to partial synteny between chromosomes 6B and 4Ug.

### 3.2 Impact of Genome Re‐Arrangements on Gene Expression and H3K4me3 Marks

Our study shows that the levels of expression and active chromatin marks for homoeologous genes located within the SRAs significantly change compared to those in the regions of the *Ae. geniculata* genome not affected by SRAs. This result suggests that the large‐scale spatial distribution of duplicated genes across the genome could affect their activity. These changes could potentially be mediated by changes in chromatin organisation in the nucleus that govern the regulation of gene expression by spatially separating facultative and constitutive heterochromatin and bringing co‐regulated genes into close proximity (Concia et al. 2020). Our results also indicate that the relative levels of expression and H3K4me3 peak areas in the duplicated homoeologous genes remain unchanged and similar to those in the genomic regions that did not experience SRAs. It is likely that changes in the functional attributes of one of the duplicate homoeologs associated with SRAs are compensated by similar changes in another gene copy, consistent with the gene dosage balance model (Conant et al. 2014). The relative levels of homoeolog expression appear to be under tight genetic control in allopolyploids, even though absolute levels of gene expression may change because of SRAs or other perturbations. These observations are consistent with the results obtained in wheat nullisomic lines, where the missing expression of deleted homoeologs is compensated by elevated expression of other homoeologs (Zhang et al. 2019). Likewise, the relative levels of homoeolog expression in the progeny of crosses between two wheat lines tend to rebalance to match the parental levels of expression by changing the expression levels of one of the homoeologs (Glombik et al. 2025). The balanced expression is likely achieved through shared *cis*‐ and *trans*‐regulatory elements (He et al. 2022).

### 3.3 Population Genomics of *Ae. geniculata* and Its Origin

Our results support the existence of two genetically differentiated populations in *Ae. geniculata*, the more genetically uniform populations north of the Bosphorus Strait, and the more highly admixed southern population (Arrigo et al. 2010). Although a previous study (Arrigo et al. 2010) did not detect significant genetic differentiation among the southern populations, using high‐density SNP‐based data, we could see clear separation among subpopulations across the southern range of the species distribution and assess the proportions of admixed ancestry in individual accessions. These analyses detected the highly differentiated populations (pop5 and pop6) of *Ae. geniculata* in Morocco growing in close geographic proximity. The prevalence of pop5 accessions in the regions with low elevation and the pop6 accessions in the regions with high elevation might be associated with their adaptation to distinct high‐ and low‐land environments. Consistently, previous studies identified substantial levels of variation in morphological and physiological traits among the *Ae. geniculata* subpopulations that could potentially be attributed to environmental adaptation (Zaharieva, Gaulin, et al. 2001; Bandou et al. 2009).

We identified the group of five *Ae. geniculata* accessions (pop8: TA10002, TA2221, TA2070, TA1848 and TA1879) genetically differentiated from the remaining accessions at *K* = 2. The accessions from pop8 show broad geographic distribution across the southern range of the species distribution, from western parts of Anatolia to western North Africa. Some of the pop8 accessions were admixed with the local populations of *Ae. geniculata* from these geographic regions. Interestingly, pop8 accessions co‐cluster with the accessions of *Ae. comosa* and *Ae. umbellulata*, the diploid donors of the M and U genomes, respectively. On the phylogenetic tree and PCA plots, pop8 showed the closest distance to the diploid ancestors. These results suggest that pop8 is closely related to the ancestral population of *Ae. geniculata*.

Our analyses identified *Ae. comosa* (TA2731) and *Ae. umbellulata* (TA2633) accessions, which have genotypes most closely related to population pop8 of *Ae. geniculata*. Both TA2731 and TA2633 are sampled in Anatolia which also has two accessions of *Ae. geniculata* (TA1848 and TA1879) from pop8. Combined, these results suggest that the origin of *Ae. geniculata* likely occurred in Anatolia by hybridization between the ancestral sympatric populations of *Ae. comosa* and *Ae. umbellulata* that had a genetic makeup closely resembling the genotypes of the modern accessions of *Ae. comosa* TA2731 and *Ae. umbellulata* TA2633. This conclusion fits well with the previous studies proposing the likely origin of *Ae. geniculata* in Turkey on the basis of AFLP/cpDNA analyses (Arrigo et al. 2010) and the geographic distribution of *Ae. comosa* and *Ae. umbellulata*.

## 4 Conclusion

Here, we presented a high‐quality genome assembly and annotation of an allotetraploid wild relative of wheat, *Ae. geniculata*. Using this resource, we characterized the evolution and origin of the M^g^ and U^g^ genomes in *Ae. geniculata* and assessed the impact of large‐scale structural rearrangements on chromosome‐level gene synteny and content, gene expression and epigenetic marks associated with active chromatin. The developed assembly provides a powerful resource for accelerating the discovery and mapping of agronomically valuable genes in *Ae. geniculata* (Stoilova and Spetsov 2006; Pradhan et al. 2012b; Gill et al. 2021; Wang et al. 2021), prioritizing its diversity for introgression into wheat (Nyine et al. 2025), and informing trait introgression strategies taking into account synteny between the genomes of *Ae. geniculata* and bread wheat.

## 5 Materials and Methods

### 5.1 *Ae. geniculata* Genome Assembly

#### 5.1.1 PacBio HiFi Library Preparation and DNA Sequencing

For PacBio HiFi sequencing, a high‐molecular‐weight (HMW) DNA was extracted from 2‐week‐old young leaf tissues of *Ae. geniculata* according to a previously described protocol (Dvorak et al. 1988). The construction and sequencing of the genomic library were performed by the KSU Integrated Genomics Facility. The DNA purity, quantity and quality were checked using NanoDrop One spectrophotometer (Thermo Fisher Scientific), Qubit 2.0 Fluorometer (Thermo Fisher Scientific), and 2200 Tape Station (Agilent), respectively. The DNA sequencing library was constructed using PacBio's SMRTbell prep kit v.3.0. The obtained library was size selected using the BluePippin system (Sage Science) with the settings recommended by PacBio. To generate HiFi reads, the SMRTbell library was sequenced with 7 SMRT cells using the Sequel IIe system (PacBio).

#### 5.1.2 Hi‐C‐Based Scaffolding

For chromosome conformation capture (Hi‐C) sequencing, the chromatin was fixed with formaldehyde in the nucleus, extracted and sent to Dovetail Genomics. To prepare Dovetail Omni‐C libraries, the fixed chromatin was digested with DNase I. Then the chromatin ends were repaired and ligated to a biotinylated bridge adapter, followed by proximity ligation of adapter‐containing ends. After proximity ligation, crosslinks were reversed, and the DNA was purified. Purified DNA was treated to remove biotin that was not internal to ligated fragments. Sequencing libraries were generated using NEBNext Ultra enzymes and Illumina‐compatible adapters. Biotin‐containing fragments were isolated using streptavidin beads before PCR enrichment of each library. The library was sequenced on an Illumina HiSeqX platform to produce approximately 30× sequence coverage.

#### 5.1.3 Genome Assembly

PacBio HiFi reads (~296 Gb) derived from the genomic DNA were *de novo* assembled using Hifiasm v0.19.5 (Cheng et al. 2021) with the default parameters. The primary contig assemblies have been submitted to Dovetail Genomics. A total of ~445 Gb Dovetail OmniC library reads have been generated by Dovetail Genomics to scaffold contigs into 14 chromosome‐scale pseudomolecules using the HiRise software (Putnam et al. 2016). A total of 453 unplaced scaffolds were assigned to a chromosome unknown (ChrUN); the assembly was updated to version Aegilops_geniculata_ksu_v1.1. To assign pseudomolecules to the chromosomes of *Ae. geniculata*, we generated low‐pass Illumina (2 × 150 bp) sequencing of *Ae. geniculata* disomic addition (DA) lines (Friebe et al. 1999). The low‐pass reads were aligned to the assembled pseudomolecules using Burrows–Wheeler Aligner (BWA) (Li and Durbin 2009). The depth of read coverage data in alignment files was calculated using SAMtools (Danecek et al. 2021) (Figure S2). The assembly was aligned to the D genome of the wheat cultivar Chinese Spring v.2.1 (CS2.1) (Zhu et al. 2021) to orient the pseudomolecules. Next, the telomeric motif‐carrying contigs at the distal ends of chromosomes 2U^g^ and 4U^g^ were manually re‐oriented to get CCCTAAA to the 5′ ends and TTTAGGG to the 3′ ends of the chromosomes. The latter two corrections re‐stored synteny to the gapless distal ends of the corresponding homoeologous telomeric regions in the long‐read‐based assembly of cv. Kariega (Athiyannan et al. 2022). At this stage, the assembly was updated to version Aegilops_geniculata_ksu_v1.2.

### 5.2 CENH3 and H3K4me3 ChIP‐Seq

Chromatin immunoprecipitation (ChIP) was performed according to the method described in Nagaki et al. (2003) standardised with wheat CENH3 antibodies (Nagaki et al. 2003; Koo et al. 2015). Antigen with the peptide sequence ‘RTKHPAVRKTKALPKK’ corresponding to the N‐terminus of wheat CENH3 was used to produce antibody utilising the custom‐antibody production facility provided by Thermo Fisher Scientific, Illinois, USA. In total, 0.396 mg of customised antibody was purified and obtained as a pellet. The pellet was dissolved in 2 mL of PBS buffer, pH 7.4, resulting in 198 ng/μL of CENH3 antibody. The specificity of the anti‐CENH3 antibody was validated using immunofluorescence assays on mitotic and meiotic chromosomes of diploid ( *Triticum monococcum* ) and hexaploid ( *Triticum aestivum* ) wheat. Nuclei were isolated from 2‐week‐old seedlings and digested with micrococcal nuclease (Sigma) to liberate nucleosomes. The digested mixture was incubated overnight with 3 μg of antibody at 4°C. Target antibodies were captured from the mixture using Dynabeads Protein G (Invitrogen, Carlsbad, CA) to obtain ChIP DNA. Mock DNA control was maintained with the input DNA following the same conditions above, without antibodies. The ChIP experiments were performed with two biological replications. Library construction was performed using the TruSeq ChIP Sample Prep Kit (Illumina, CA) according to the manufacturer's instructions, and the libraries were sequenced using the NovoSeq X system with a 2 × 150 bp sequencing run.

### 5.3 Genome Annotation

#### 5.3.1 RNA‐Seq Data Generation

For RNA sequencing, samples from the seedling stage leaf, seedling stage root, flag leaf, leaf sheath, whole spike, pre‐anthesis spike and stem in three biological replicates were collected. The biological replicates from the same tissues were combined in equal amounts, ground into powder and used to extract RNA with TRIzol Reagent (Thermo Fisher Scientific). The assessment of RNA quality was performed using Agilent 2100 Bioanalyzer (Agilent). RNA‐Seq libraries were prepared using the Illumina Stranded mRNA Ligation prep kit at the KSU IGF and sequenced on an Illumina NovaSeq S4 flow cell (2 × 150 bp) to generate ~1 billion paired‐end reads totaling 322.5 Gb (Table S3) at the University of Kansas Medical Center. After quality control and adapter trimming using Cutadapt (Martin 2011), the reads were aligned to *Ae. geniculata* genome assembly using the splicing‐aware STAR aligner (Dobin et al. 2013).

#### 5.3.2 PacBio Iso‐Seq and Transcriptome Clustering

The total RNA extracted from the seedling stage root and pre‐anthesis spike was used to perform Iso‐Seq. Iso‐Seq libraries were prepared using the Iso‐Seq express oligo kit, NEBNext Single Cell Low Input cDNA synthesis and Amplification Module and SMRTbell prep kit v.3.0 (PacBio) and sequenced on 2 SMRT cells using a PacBio Sequel IIe system at the KSU IGF. Obtained 18 Gb HiFi reads were Poly(A)/primers trimmed and clustered to 752.7 Mb high‐quality transcripts (Table S4) using the PacBio SMRT Link software v11.0.

#### 5.3.3 Annotation of Gene Models

Gene models were annotated using the evidence‐based EviAnn annotation pipeline (Zimin et al. 2025). EviAnn predicts protein‐coding regions and long non‐coding RNA (lncRNA) annotations using alignments of RNA‐seq, transcript assemblies, and proteins from the related species. The RNA‐seq and Iso‐Seq data generated for *Ae. geniculata* along with 913 360 unique protein sequences of the related *Triticeae* species (*Aegilops*, *Dasypyrum*, *Thinopyrum* and *Triticum*) downloaded from the NCBI protein database have been used for predicting the protein‐coding and lncRNA genes. NLRs were annotated using NLRtracker (v1.0.3) with default settings (Kourelis et al. 2021). The predicted gene models were named with AGAT (Dainat 2020) using the following naming scheme, AegTA2899_1M01G00000000100, where the AegTA2899 corresponds to the species and accession name, 1M01—chromosome name and version of annotation, G00000000100—gene number. Annotation of transposable elements was performed using RepeatMasker v. 4.1.4, the repetitive element database from GIRI (trip‐db_complete_Rel‐19 library). The quality of genome annotation was assessed using BUSCO (Simão et al. 2015).

The genome ideogram was visualised using the karyoploteR (Gel and Serra 2017). The ChIPseeker (Yu et al. 2015) package was used to identify the genomic features associated with the ChIP‐Seq enriched regions.

### 5.4 Analysis of Genome Synteny

To identify homoeologs in the M^g^ and U^g^ genomes, reciprocal best blast hit (RBH) analysis was performed iteratively until no additional RBHs were detected. Each cycle consisted of five steps:

1. A BLASTn index was created using the gene sequences from each subgenome.
2. The genes from each genome were compared against the other in both directions (A → B and B → A).
3. Selection of Best Hits (BHs): From each BLASTn output, the best hits were selected on the basis of the following criteria: The proportion of target gene coverage ≥ 90%, sequence identity ≥ 90% and e‐value ≤ 1 × 10E−5.
4. RBHs were determined by retaining only those gene pairs that were each other's top match in both comparisons.
5. It is common to have multiple hits that satisfy the cutoff parameters. In the first round, some genes may be paired with their closest match, but other valid pairs might not be detected because of the presence of these initial matches. By removing the genes identified in the first cycle and repeating the process, we increase the likelihood of finding additional homoeologous pairs that may have been overlooked in earlier rounds. Genes identified as RBHs were removed from the fasta files, and the process was repeated again until no new RBHs were found.

Syntenic blocks were identified using the homoeologous gene pairs obtained from the RBH analysis as input for MCScanX v.1.0.0 (Wang et al. 2012), which was run with default parameters. The resulting collinear blocks were visualised using SynVisio (Bandi and Gutwin 2020). On the basis of the collinearity data generated by MCScanX, we identified the collinear blocks located within the rearranged regions. When multiple small collinear blocks were located near one another on the same chromosome, they were merged into a larger block to better represent the full extent of the rearrangement.

### 5.5 H3K4me3 ChIP‐Seq Data Analysis

Two ChIP‐seq replicates were generated from leaf tissue of *Ae. geniculata* on the seedling stage leaf tissue, targeting the H3K4me3 histone modification. After quality control, peak calling was performed using the callpeak function from MACS3 (v3.0.1) for each replicate independently (Zhang et al. 2008). Following this, the bdgcmp ‐m FE function in MACS3 was used to compute the fold enrichment (FE) over control peaks. To ensure the reliability of peak identification, only reproducible peaks with IDR < 0.1 across replicates were retained. IDR filtering was conducted using IDR v2.0.3 (Li et al. 2011), and only peaks passing this threshold in both replicates were used for subsequent analysis.

To measure the distribution of H3K4me3 enrichment around genes, we used a custom R script to generate a weighted profile matrix considering 2 kb upstream, the gene body, and 2 kb downstream of each gene. The fold enrichment (FE) values from the ChIP‐seq signal were used to quantify H3K4me3 coverage across these regions. For each gene, the area under the FE curve was calculated to represent the total H3K4me3 signal across the defined region.

To evaluate differential histone enrichment between homoeologous genes, we computed the H3K4me3 Peak Homoeologous Bias (HPHB) as: HPHB = log2 (Area_geneM+1E−6_/Area_geneU+1E−6_). The small constant was added to avoid undefined values in cases where the signal was absent in one or both homoeologous. Positive HPHB values indicate stronger H3K4me3 enrichment in the gene from the M^g^ genome, whereas negative values indicate enrichment in the gene from the U^g^ genome.

### 5.6 Gene Expression Analyses

Gene expression levels were quantified by pseudo‐aligning reads to the *Ae. geniculata* reference genome using Kallisto (v0.48.0) (Bray et al. 2016). Isoform‐level abundances were summarised to gene‐level estimates with the tximport package (v1.34.0). Expression values were normalised as Transcripts Per Million (TPM), and genes were considered expressed if their cumulative TPM across all tissues was at least 10 TPM. To estimate overall expression per gene, we defined a global TPM as the mean TPM across tissues in which the gene was expressed.

#### 5.6.1 Homoeologous Gene Expression Analyses

To investigate the dynamics of homoeologous gene expression, we calculated the homoeolog expression bias (HEB) for each expressed dyad across tissues: HEB_i_ = log2(TPM_Mi_/TPM_Ui_), where HEB_i_ is the homoeolog expression bias for the dyad *i*, and TMP_M_ and TMP_U_ are the non‐normalised expression values for homoeologs *i* in subgenomes M^g^ and U^g^, respectively. HEB_*i*_ = 0 indicates no bias, positive values indicate higher expression of homoeolog M_*i*_, and negative values indicate higher expression of homoeolog U_*i*_. In cases when only one of the genes in a dyad is expressed and another one is silenced, we used TPM values of 10E−6 for a non‐expressed copy. Absolute TPM values were normalised for each homoeologous gene within the dyad as follows: normTPM_M = TPM_M/(TPM_M + TPM_U), normTPM_U = TPM_U/(TPM_M + TPM_U). This ensured that the expression of each homoeolog within the dyad was represented as a proportion of the total expression for that gene pair, avoiding the impact of absolute values on downstream analyses. Using the normalised expression values, the dyads were classified into three categories on the basis of the expected M^g^‐genome/U^g^‐genome expression ratio values: (1) balanced dyads with a 0.5 M/0.5 U ratio, (2) M^g^‐dominant with a 1.0 M/0.0 U ratio, and (3) U^g^‐ dominant 0.0 M/1.0 U ratio. Each dyad was assigned to one of the three expression categories on the basis of the shortest Euclidean distance to the expected ratios.

#### 5.6.2 Variation in Homoeologous Expression Bias Across Tissues

To investigate the homoeologous expression bias patterns across tissues, we classified the dyads as stable, dynamic or intermediate. The distance between the global expression of a gene and its expression in individual tissues was calculated. The average of these distances was defined as the dyad's mean distance. Dyads were then ranked on the basis of their dyad mean distance, comparing the distribution of dyads in terms of their deviation from the expected expression pattern. percentile_*i* = truncate(rank(cmd_*i*)/length(CMD)), where cmd_*i* is the Coefficient of Mean Distance (CMD) for the *i*th dyad, and CMD is the vector containing the mean distances of all dyads. The deciles were then classified as stable, dynamic and intermediate, with the first two considered stable, the last two considered dynamic and the remaining classified as intermediate.

### 5.7 Analyses of Genetic Diversity

For SNP genotyping, the published (Adhikari et al. 2023) raw reads derived from the 936 plants of 21 *Aegilops* species (Table S19) were mapped to the Aegilops_geniculata_ksu_v1.3 reference using BWA (Li and Durbin 2009). Further, the BAM files were filtered to remove the PCR duplicates and non‐uniquely mapped reads. The variant calling was performed using the GATK's HaplotypeCaller (McKenna et al. 2010). The GVCF files were combined by the CombineGVCFs and processed by the GenotypeGVCFs to generate a multisample VCF file with ~16 million genomic variants. Then we extracted ~6.6 million M^g^ genome SNPs from 337 accessions and ~9.6 million U^g^ genome SNPs from 566 accessions with SNP calls supported by at least two reads. We kept only those SNPs that had calls in more than 50% of the accessions with the minor allele frequency greater than 5%. This filtering was performed using PLINK (Purcell et al. 2007) with ‐‐maf 0.05 ‐‐geno 0.50 parameters. The resulting SNPs set included 44 861 SNPs with a total genotyping rate of 0.67 for the M^g^ genome dataset and 51 060 SNPs with a total genotyping rate of 0.72 in the U^g^ genome dataset.

### 5.8 Depth of Reads Coverage Analysis

The BAM files generated for *Aegilops* species and genetic stocks with the additions of *Ae. geniculata* chromosomes were also used to calculate the mean depth of read coverage in 50 Mb windows across the genome. Further, the coverage was normalised to the median and used to analyse coverage across the chromosomes of all individuals. The coverage data generated for *Aegilops* species was used to validate the ploidy level of the analysed accessions. It is expected that the *Ae. comosa* accessions should have higher coverage in the M^g^ genome, whereas the *Ae. umbellulata* accessions should have higher coverage in the U^g^ genome. Plants labelled as *Ae. geniculata* were required to have coverage in both M^g^ and U^g^ genomes. The lines with inconsistencies in coverage data were marked as mislabeled. The coverage data generated for genetic stocks with the additions of *Ae. geniculata* chromosomes were used to assign pseudomolecules to the chromosomes of the M^g^ and U^g^ genomes (Figure S2).

### 5.9 Principal Component Analysis (PCA)

The principal components were calculated in PLINK with the ‐‐pca parameter, resulting in a relationship matrix of 20 components plotted using a custom R script. The first two principal components explaining 81% of the differences between the plants were plotted to show the distribution of genomic components of the M genome‐carrying polyploids in relation to the M genome donor species *Ae. comosa*, as well as U genome‐carrying polyploids in relation to the U genome donor species *Ae. umbellulata* (Figure S10).

### 5.10 Phylogenetic Analyses

For the phylogenetic analyses, we calculated a matrix of pairwise genetic distances between individuals in the SNP dataset, on the basis of Identity‐By‐State (IBS) allele count using PLINK with the “‐‐distance square ibs allele‐ct” parameters. The obtained distance matrix was used to generate an unrooted neighbour‐joining (NJ) tree in FigTree v1.4.5.

### 5.11 ADMIXTURE Analyses

Ancestry proportions were analysed using the maximum likelihood‐based ADMIXTURE software (Alexander et al. 2009). ADMIXTURE was run for a range of clusters (*K*) from 1 to *N* (where *N* was the number of species in the dataset), with 10 replicates performed for each *K* value. The best‐supported number of clusters (K) was determined using the cross‐validation procedure for haploids (‐‐haploid = “*” ‐‐cv), where the *K* with the lowest cross‐validation error was selected as optimal. The ancestry proportions of individuals for the selected *K* were plotted using a custom R script.

## Author Contributions

U.Y.: formal analysis, software and visualisation. G.R.C.: formal analysis, software and visualisation. R.S.: investigation. E.L.: formal analysis, software and visualisation. J.W.R.: resources. A.V.Z.: formal analysis and software. A.A.: investigation and resources. D.‐H.K.: investigation, resources and supervision. E.A.: supervision, conceptualization, methodology, writing – review and editing, project administration and funding acquisition.

## Conflicts of Interest

The authors declare no conflicts of interest.

## Supporting information


**Table S1:** General statistics of *Ae. geniculata* HiFi reads and genome assembly.
**Table S2:**. Density peaks obtained for CENH3 ChIP‐seq reads and centromeric repeats.
**Table S3:** *Ae. geniculata* RNA‐seq Illumina raw paired‐end (2 × 150 bp) reads and Trinity assembled transcripts.
**Table S4:** *Ae. geniculata* Iso‐Seq HiFi reads and clustered HQ transcripts.
**Table S5:**. General statistics of *Ae. geniculata* annotation using EviAnn.
**Table S6:**. BUSCO assessment of the *Aegilops geniculata* genome assembly*.
**Table S7:**. Annotation of TE families in the M and U genomes of *Ae. geniculata*.
**Table S8:** Differences in the lengths of chromosomes and total TE content between the M^g^ and U^g^ genomes.
**Table S9:** Number of homoeologous gene pairs identified on the basis of the reciprocal best blast hit analyses of annotated gene models.
**Table S10:**. Boundaries of 13 structural re‐arrangements detected by comparing the M^g^ and U^g^ genomes of *Ae. geniculata*.
**Table S11:**. Proportion of genes from the NLR family of immune receptors relative to the total number of genes for each chromosome.
**Table S12:** Mapping candidate resistance gene intervals to the *Ae. geniculata* genome.
**Table S13:** Summary of RNA‐seq data and expressed genes across different tissues in *Ae. geniculata*.
**Table S14:** Distribution of expressed genes across tissues. Gene is considered not expressed if it shows TPM < 1 in any given tissue.
**Table S15:**. Grouping genes on the basis of the coefficient of variation (CV) in gene expression across seven tissues.
**Table S16:**. Number of homoeologous genes from dyads classified according to gene expression CV across tissues.
**Table S17:** Homoeolog expression classification per tissue.
**Table S18:** List of *Aegilops* species used for data curation.
**Table S19:**. General statistics* of the genomic variants in the 21 *Aegilops* species genotyped on the Aegilops_geniculata_ksu_v1.3 reference genome.
**Table S20:**. Data curation for species assignment using SNPs from the panel of 21 *Aegilops* species.
**Table S21:** List of *Ae. umbellulata, Ae. comosa* and *Ae. geniculata* accessions used for diversity analyses.
**Table S22:**. General statistics of the SNPs called in the M and U genomes by mapping reads from *Ae. umbellulata, Ae. comosa* and *Ae. geniculata* to Aegilops_geniculata_ksu_v1.3 reference genome.
**Figure S1:**. OmniC reads link density heatmap in the HiRise scaffolded *Ae. geniculata* pseudomolecules. The intensity of colours on the heatmap reflects the frequency of contacts on the Hi‐C map.
**Figure S2:**. Heatmap of the depth of read coverage derived from genomic DNA of the *T. aestivum* —*Ae. geniculata* disomic addition (DA) lines. The reads were aligned to the genomes of CS2.1 and *Ae. geniculata*. The depth was calculated for 50Mbp windows (coloured verticals lines) and normalised to a median. The intensity of red and blue colours reflect increase or decrease in the depth of read coverage, respectively, relative to the genome‐wide median coverage shown in white colour.
**Figure S3:**. Centromere positions on the basis of the CenH3 ChIP‐seq data. The red and blue dashed lines correspond to results obtained using ChIP‐seq biological replicates. The dots mark the summit of each peak.
**Figure S4:**. Ideogram of the Aegilops_geniculata_ksu_v1.3.fna assembly pseudomolecules with telomeric TTTAGGG (forward−blue/reverse complement−cyan, 10tandems − long/5tandems − short columns), centromeric CENH3 (orange), CRW2_LG_3L(pink), Quinta_LTRL3(green), Quinta_R5(grey) motifs density, mean depth of reads coverage (dot = 1 Mb window) for the back aligned TA2899 HiFi reads (grey), TA2899 CENH3 ChIP‐seq reads (salmon red) and gaps between contigs (black columns). Length of the pseudomolecules given in Mbp in the *x*‐axis, the HiFi reads depth *y*‐axis, max is 20×, and for CENH3 ChIP‐seq reads, the y‐axis max is 60×.
**Figure S5:**. Detection of duplicated homoeologs and U^g^ and M^g^ genome‐specific sets of genes by reciprocal best blast hit analysis.
**Figure S6:**. Dot plots show alignment of U^g^ genome chromosomes with homoeologous chromosomes from M^g^ and wheat D genome.
**Figure S7:**. Dot plots show alignment of 4 U^g^ chromosomes with chromosomes from the M^g^ genome.
**Figure S8:**. Distribution of homoeologous expression bias (HEB) values calculated using gene expression data from different tissues.
**Figure S9:**. PCA analyses of *Aegilops* species in the diversity panel from the KSU WGRC collection. This analysis was used to exclude misclassified accessions. Only those accessions that co‐cluster with samples labelled as *Ae. geniculata*, *Ae. comosa* or *Ae. umbellulata* were retained.
**Figure S10:**. PCA analyses of polyploids carrying the M and/or U genomes was aimed at evaluating their clustering patterns with the diploid genome donors *Ae. comosa* (M) and *Ae. umbellulata* (U).

## Data Availability

All genome sequencing data generated for this project are available under NCBI Bioproject PRJNA1257951 (https://www.ncbi.nlm.nih.gov/). The whole genome sequence of *Ae. geniculata* and its annotation are deposited to NCBI under accession JBQUYH000000000, BioSample SAMN48921477.
