# Supplementary material for: The Evolution and Origin of Allotetraploid Aegilops geniculata Revealed by the Homoeolog‐Resolved Genome Assembly
Source: Plant Biotechnol J. 2025 Nov 10;24(3):1866–84. doi: 10.1111/pbi.70456 (PMC12946516; doi:10.1111/pbi.70456)
Supplement: Supplementary file 1 — Table S1: General statistics of Ae. geniculata HiFi reads and genome assembly. Table S2:. Density peaks obtained for CENH3 ChIP‐seq reads and centromeric repeats. Table S3: Ae. geniculata RNA‐seq Illumina raw paired‐end (2 × 150 bp) reads and Trinity assembled transcripts. Table S4: Ae. geniculata Iso‐Seq HiFi reads and clustered HQ transcripts. Table S5:. General statistics of Ae. geniculata annotation using EviAnn. Table S6:. BUSCO assessment of the Aegilops geniculata genome assembly*. Table S7:. Annotation of TE families in the M and U genomes of Ae. geniculata. Table S8: Differences in the lengths of chromosomes and total TE content between the Mg and Ug genomes. Table S9: Number of homoeologous gene pairs identified on the basis of the reciprocal best blast hit analyses of annotated gene models. Table S10:. Boundaries of 13 structural re‐arrangements detected by comparing the Mg and Ug genomes of Ae. geniculata. Table S11:. Proportion of genes from the NLR family of immune receptors relative to the total number of genes for each chromosome. Table S12: Mapping candidate resistance gene intervals to the Ae. geniculata genome. Table S13: Summary of RNA‐seq data and expressed genes across different tissues in Ae. geniculata. Table S14: Distribution of expressed genes across tissues. Gene is considered not expressed if it shows TPM < 1 in any given tissue. Table S15:. Grouping genes on the basis of the coefficient of variation (CV) in gene expression across seven tissues. Table S16:. Number of homoeologous genes from dyads classified according to gene expression CV across tissues. Table S17: Homoeolog expression classification per tissue. Table S18: List of Aegilops species used for data curation. Table S19:. General statistics* of the genomic variants in the 21 Aegilops species genotyped on the Aegilops_geniculata_ksu_v1.3 reference genome. Table S20:. Data curation for species assignment using SNPs from the panel of 21 Aegilops species. Table S21: List of Ae. umb [file PBI-24-1866-s001.docx]

SUPPLEMENTARY INFORMATION

***The evolution and origin of allotetraploid Aegilops geniculata revealed by the homoeolog-resolved genome assembly.***

Ural Yunusbaev^1,2^, Gabriela Romero Campos^1,2^, Sathish Raj Rajendran^1,2^, Evgenii Liakh^1,2^, John W. Raupp^1,2^, Alexey V. Zimin^3^, Alina Akhunova^1,4^, Dal-Hoe Koo^1,2^, Eduard Akhunov^1,2^

^1^ Department of Plant Pathology, Kansas State University, Manhattan, USA

^2^ Wheat Genetics Resource Center, Kansas State University, Manhattan, USA

^3^ Department of Biomedical Engineering, Johns Hopkins University, Baltimore, MD, USA

^4^ Integrated Genomics Facility, Kansas State University, Manhattan, USA

Corresponding author: eakhunov@ksu.edu

**Supplementary Tables**

**Table S1**. General statistics of *Ae. geniculata* HiFi reads and genome assembly.

| Parameter | Value |
| --- | --- |
| HiFi reads |  |
| number | 16,404,208 |
| total size (bp) | 295,903,998,027 |
| mean length (bp) | 18,038.30 |
| coverage (x) | ~30 |
| Hifiasm contigs |  |
| number | 1,442 |
| total size (bp) | 8,114,126,630 |
| mean length (bp) | 5,626,995 |
| longest (bp) | 60,506,019 |
| shortest (bp) | 17,930 |
| N50 | 14,825,337 |
| Gaps | 0 |
| HiRise scaffolds |  |
| number | 467 |
| total size (bp) | 8,114,226,130 |
| pseudomolecules | 14 |
| unplaced | 453 |
| Gaps | 981 |

**Table S2.** Density peaks obtained for CENH3 ChIP-seq reads and centromeric repeats.

| Chromosome | Length, bp | Centromere | | Centromeric repeats | |
| --- | --- | --- | --- | --- | --- |
|  |  | Start, Mb | End, Mb | Start, Mb | End, Mb |
| 1M | 509,995,657 | 190 | 196 | 180 | 200 |
| 1U | 466,560,248 | 171 | 175 | 160 | 230 |
| 2M | 604,824,310 | 168 | 175 | 165 | 190 |
| 2U | 611,755,209 | 132 | 138 | 130 | 190 |
| 3M | 652,463,001 | 275 | 279 | 170 | 185 |
| 3U | 539,506,882 | 149 | 154 | 150 | 160 |
| 4M | 629,564,697 | 248 | 253 | 235 | 250 |
| 4U | 530,872,227 | 68 | 73 | 60 | 80 |
| 5M* | 575,558,482 | 150 | 153 | 145 | 190 |
|  |  | 174 | 178 |  |  |
| 5U | 583,471,674 | 160 | 165 | 160 | 180 |
| 6M | 455,192,259 | 192 | 196 | 190 | 200 |
| 6U | 615,817,754 | 273 | 279 | 255 | 285 |
| 7M | 672,203,257 | 333 | 339 | 330 | 345 |
| 7U | 625,570,992 | 146 | 151 | 120 | 160 |
| Unknown | 40,914,781 |  |  |  |  |

*Two CENH3 peaks are detected

**Table S3.** *Ae. geniculata* RNA-seq Illumina raw paired-end (2 x 150 bp) reads and Trinity assembled transcripts.

| Tissue | Raw PE reads | | | Assembled transcripts | | | | |
| --- | --- | --- | --- | --- | --- | --- | --- | --- |
|  | number | sum length | length | number | sum length | min | avg | max |
| Seedling stage leaf | 152,009,624 | 45,906,906,448 | 300 |  |  |  |  |  |
| Seedling stage root | 174,134,498 | 52,588,618,396 | 300 |  |  |  |  |  |
| Flag leaf | 121,445,594 | 36,676,569,388 | 300 |  |  |  |  |  |
| Leaf sheath | 140,511,307 | 42,434,414,714 | 300 |  |  |  |  |  |
| Whole spike | 159,867,888 | 48,280,102,176 | 300 |  |  |  |  |  |
| Pre-anthesis spikes | 158,875,407 | 47,980,372,914 | 300 |  |  |  |  |  |
| Stem | 160,985,095 | 48,617,498,690 | 300 |  |  |  |  |  |
| Merged | 1,067,829,413 | 322,484,482,726 | 300 | 594,583 | 958,856,547 | 180 | 1,612.70 | 34,240 |

**Table S4.** *Ae. geniculata* Iso-Seq HiFi reads and clustered HQ transcripts.

| Tissue | HiFi reads | | | | | Clustered HQ transcripts | | | | |
| --- | --- | --- | --- | --- | --- | --- | --- | --- | --- | --- |
|  | number | sum length | min | avg | max | number | sum length | min | avg | max |
| Pre-anthesis spike | 2,181,231 | 7,575,006,492 | 156 | 3,472.80 | 21,582 | 98,233 | 335,913,193 | 81 | 3,419 | 12,566 |
| Seedling root | 3,075,852 | 10,460,509,819 | 121 | 3,400.80 | 21,704 | 126,113 | 416,852,472 | 67 | 3,305 | 13,429 |
| Merged | 5,257,083 | 18,035,516,311 | 121 | 3,436.80 | 21,704 | 224,346 | 752,765,665 | 67 | 3,355 | 13,429 |

**Table S5.** General statistics of *Ae. geniculata* annotation using EviAnn.

| **Annotated genome features** | **Number of features** |
| --- | --- |
| Number of genes | 74,143 |
| Number of protein coding genes | 72,091 |
| Number of processed pseudo gene transcripts | 1,862 |
| Number of processed pseudo genes | 1,860 |
| Number of transcripts | 128,205 |
| Number of long non-coding RNAs | 2,611 |
| Number of distinct proteins | 111,288 |

**Table S6.** BUSCO assessment of the *Aegilops geniculata* genome assembly*.

| **BUSCO results** | **n** | **%** |
| --- | --- | --- |
| Complete genes (C) | 4856 | **99.2** |
| Complete and single-copy (S) | 339 | 6.9 |
| Complete and duplicated (D) | 4517 | 92.3 |
| Fragmented (F) | 10 | 0.2 |
| Missing (M) | 30 | 0.6 |
| Total groups searched | 4896 | 100 |

*BUSCO ver. 4.0.5 was used with the eukaryota_odb10 database created on 2020-09-10.

**Table S7.** Annotation of TE families in the M and U genomes of *Ae. geniculata.*

|  | **M genome** | | **U genome** | | **Fold change (U^g^/M^g^)** | **χ-test** | |
| --- | --- | --- | --- | --- | --- | --- | --- |
| **Families^*^** | **Count** | **Length, bp** | **Count** | **Length, bp** |  | **p-value** | **p-value (BH)** |
| **Class I** |  |  |  |  |  |  |  |
| RIC | 2 | 113 | 2 | 106 | 0.94 | 8.39E-01 | 8.39E-01 |
| RII | 122 | 6,524 | 122 | 6,352 | 0.97 | 5.82E-01 | 6.07E-01 |
| RIJ | 61 | 4,839 | 93 | **5,366** | 1.11 | 1.70E-12 | 2.13E-12 |
| RIL | 2,191 | 1,165,239 | 2,409 | **1,483,177** | 1.27 | 0.00E+00 | 0.00E+00 |
| RIP | 16 | 964 | 7 | 376 | 0.39 | 1.66E-53 | 2.44E-53 |
| RIR | 646 | 534,002 | 809 | **630,032** | 1.18 | 0.00E+00 | 0.00E+00 |
| RIX | 54,001 | 40,877,505 | 53,774 | **41,736,684** | 1.02 | 0.00E+00 | 0.00E+00 |
| RLA | 5 | 396 | 5 | 355 | 0.9 | 3.19E-01 | 3.46E-01 |
| RLC | 116,808 | 1,162,040,186 | 95,440 | 837,313,979 | 0.72 | 0.00E+00 | 0.00E+00 |
| RLG | 236,049 | 1,591,857,674 | 243,288 | **1,870,695,895** | 1.18 | 0.00E+00 | 0.00E+00 |
| RLH | 12 | 647 | 13 | **766** | 1.18 | 1.18E-04 | 1.40E-04 |
| RLX | 41,845 | 109,785,325 | 37,954 | 71,077,828 | 0.65 | 0.00E+00 | 0.00E+00 |
| RSX | 18,759 | 6,794,453 | 17,697 | 6,183,880 | 0.91 | 0.00E+00 | 0.00E+00 |
| **Class II** |  |  |  |  |  |  |  |
| DHH | 32,548 | 8,281,484 | 32,391 | **8,570,856** | 1.03 | 0.00E+00 | 0.00E+00 |
| DTA | 2,451 | 549,283 | 2,788 | **611,713** | 1.11 | 0.00E+00 | 0.00E+00 |
| DTB | 71 | 4,335 | 65 | 3,922 | 0.9 | 3.84E-03 | 4.36E-03 |
| DTC | 217,796 | 361,692,927 | 202,847 | 328,449,153 | 0.91 | 0.00E+00 | 0.00E+00 |
| DTH | 28,812 | 13,964,817 | 29,831 | **14,295,070** | 1.02 | 0.00E+00 | 0.00E+00 |
| DTM | 47,842 | 18,828,459 | 45,152 | 18,354,652 | 0.97 | 2.99E-243 | 4.67E-243 |
| DTP | 3 | 194 | 6 | **391** | 2.02 | 8.95E-18 | 1.24E-17 |
| DTT | 52,535 | 7,960,905 | 50,324 | 7,645,631 | 0.96 | 2.96E-15 | 3.90E-15 |
| DTX | 2,943 | 528,942 | 3,222 | **761,115** | 1.44 | 0.00E+00 | 0.00E+00 |
| DXX | 4,064 | 959,274 | 4,380 | **1,080,748** | 1.13 | 0.00E+00 | 0.00E+00 |
| **Unclassif** |  |  |  |  |  |  |  |
| NUL | 648 | 148,850 | 1,027 | **203,824** | 1.37 | 0.00e+00 | 0.00e+00 |
| XXX | 33,975 | 16,221,425 | 32,743 | 13,590,031 | 0.84 | 0.00e+00 | 0.00e+00 |
| **TOTAL** | 894,205 | 3,342,208,762 | 856,389 | 3,222,701,902 |  |  |  |

^a^ RLG,1,Class I: LTR-Gypsy

DTC,2,Class II: CACTA

RLC,1,Class I: LTR-Copia

RIX,1,Class I: LINEs

DTT,2,Class II: Mutator

DTM,2,Class II: Mariner/Tc1

RLX,1,Class I: LTR-unclassified

XXX,NA,Unclassified/ambiguous

DHH,2,Class II: Helitron

DTH,2,Class II: Helitron

RSX,1,Class I: SINE-unclassified (often SINEs)

DXX,2,Class II: unknown DNA TE

DTX,2,Class II: DNA TE unclassified

DTA,2,Class II: hAT superfamily

RIL,1,Class I: LINE superfamily

NUL,NA,Likely unknown or placeholder

RIR,1,Class I: LINE

RII,1,Class I: LINE

RIJ,1,Class I: LINE

DTB,2,Class II: PIF/Harbinger

RLH,1,Class I: Penelope-like elements (PLEs)

RIP,1,Class I: Possibly non-LTR retroelement

RLA,1,Class I: Retrovirus-like

DTP,2,Class II: piggyBac

RIC,1,Class I: LINE

**Table S8:** Differences in the lengths of chromosomes and total TE content between the M^g^ and U^g^ genomes.

| **M^g^ chr.** | **M^g^ chr. length (bp)** | **U^g^ chr.** | **U^g^ chr. length (bp)** | **Length diff. (U-M)*** | **TE length diff. (U-M)*** |
| --- | --- | --- | --- | --- | --- |
| 1M | 509,995,657 | 1U | 466,560,248 | -43,435,409 | -42,697,239 |
| 2M | 604,824,310 | 2U | 611,755,209 | 69,30,899 | 11,254,741 |
| 3M | 652,463,001 | 3U | 539,506,882 | -112,956,119 | -91,461,222 |
| 4M | 629,564,697 | 4U | 530,872,227 | -98,692,470 | -100,744,140 |
| 5M | 575,558,482 | 5U | 583,471,674 | 7,913,192 | 9,043,953 |
| 6M | 455,192,259 | 6U | 615,817,754 | 160,625,495 | 136,340,042 |
| 7M | 672,203,257 | 7U | 625,570,992 | -46,632,265 | -41,242,995 |

*Negative values indicate that the length of chromosomes or combined length of TEs is higher in the M^g^ genome than in the U^g^ genome.

**Table S9:** Number of homoeologous gene pairs identified based on the reciprocal best blast hit analyses of annotated gene models.

| **Genome I** | **Genome II** | **Syntenic genes** |
| --- | --- | --- |
| D genome (wheat) | *Ae. comosa* | 21280 |
| *Ae. comosa* | *Ae. gen.* - M genome | 22548 |
| *Ae. gen.* - M genome | *Ae. gen.* - U genome | 21167 |
| *Ae. gen.* - U genome | *Ae. umbellulata* | 22608 |
| *Ae. umbellulata* | D genome (wheat) | 21902 |

**Table S10.** Boundaries of 13 structural re-arrangements detected by comparing the M^g^ and U^g^ genomes of *Ae. geniculata.*

| **Chr A** | **Chr B** | **Start A** | **Start B** | **End A** | **End B** | **Total # of syntenic blocks merged** | **Total # of genes** |
| --- | --- | --- | --- | --- | --- | --- | --- |
| Chr1M | Chr4U | 459756332 | 479391653 | 509930640 | 530575379 | 6 | 964 |
| Chr2M | Chr4U | 506475599 | 452816144 | 532754791 | 478084676 | 5 | 454 |
| Chr3M | Chr7U | 98946 | 565533344 | 72905904 | 625534552 | 12 | 912 |
| Chr4M | Chr4U | 1818029 | 1248925 | 331232572 | 366539292 | 51 | 2078 |
| Chr4M | Chr4U | 564171162 | 363030918 | 599847759 | 406222173 | 3 | 436 |
| Chr4M | Chr5U | 32177 | 543976913 | 563884026 | 583309053 | 4 | 750 |
| Chr4M | Chr6U | 306601580 | 172622943 | 529174831 | 613534755 | 21 | 1980 |
| Chr5M | Chr6U | 537080231 | 606337544 | 542251853 | 615670091 | 2 | 70 |
| Chr6M | Chr4U | 82241 | 279881281 | 58700507 | 351188759 | 9 | 524 |
| Chr6M | Chr6U | 45564749 | 264516 | 455164935 | 394676478 | 69 | 3368 |
| Chr7M | Chr2U | 600202445 | 123689 | 671721810 | 37284133 | 2 | 24 |
| Chr7M | Chr4U | 614467063 | 406462419 | 670930748 | 452423818 | 4 | 602 |
| Chr7M | Chr7U | 1790380 | 101919 | 249467098 | 564272027 | 27 | 2472 |

**Table S11.** Proportion of genes form the NLR family of immune receptors relative to the total number of genes for each chromosome.

| **Chromosome** | **Number of NLRs** | **Num. Genes** | **Proportion** |
| --- | --- | --- | --- |
| Chr1M | 79 | 4432 | 1.78% |
| Chr1U | 63 | 3619 | 1.74% |
| Chr2M | 89 | 6034 | 1.47% |
| Chr2U | 79 | 5573 | 1.42% |
| Chr3M | 82 | 5640 | 1.45% |
| Chr3U | 50 | 4511 | 1.11% |
| Chr4M | 69 | 5371 | 1.28% |
| Chr4U | 121 | 5460 | 2.22% |
| Chr5M | 80 | 5571 | 1.44% |
| Chr5U | 70 | 5621 | 1.25% |
| Chr6M | 59 | 3474 | 1.70% |
| Chr6U | 38 | 4549 | 0.84% |
| Chr7M | 174 | 5898 | 2.95% |
| Chr7U | 119 | 5943 | 2.00% |

**Table S12:** Mapping candidate resistance gene intervals to the *Ae. geniculata* genome.

| **Locus** | **Chr.** | **Markers** | **Start - end (bp)** | **Candidate R genes** |
| --- | --- | --- | --- | --- |
| *Lr9* | 4Ug | Cloned gene | 461982130-461991457 | MAKER_6U01G00001710300 |
| *Lr76_Yr70* | 5Ug | TraesCS5D02G005300 - TraesCS5D02G012500 | 13151521 -26730738 | AegTA2899_5U01G00000016800 |
|  |  |  |  | AegTA2899_5U01G00000017100 |
|  |  |  |  | AegTA2899_5U01G00000017400 |
|  |  |  |  | AegTA2899_5U01G00000017500 |
|  |  |  |  | AegTA2899_5U01G00000017700 |
|  |  |  |  | AegTA2899_5U01G00000019100 |
|  |  |  |  | AegTA2899_5U01G00000020400 |
|  |  |  |  | AegTA2899_5U01G00000021200 |
|  |  |  |  | AegTA2899_5U01G00000021300 |
|  |  |  |  | AegTA2899_5U01G00000023600 |
|  |  |  |  | AegTA2899_5U01G00000025100 |
|  |  |  |  | AegTA2899_5U01G00000025500 |
|  |  |  |  | AegTA2899_5U01G00000028900 |
|  |  |  |  | AegTA2899_5U01G00000032300 |
|  |  |  |  |  |
| *Lr57* | 5Mg | TraesCS5D02G006400 - TraesCS5D02G005600 | 6509469 - 8074638 | AegTA2899_5M01G00000006500 |
|  |  |  |  |  |
|  |  |  |  |  |
| *Yr40* | 5Mg | TraesCS5D02G014100 - TraesCS5D02G016600 | 8524932 - 9615243 | AegTA2899_5M01G0000001150 |
|  |  |  |  | *MAKER_5M01G00000038100* |
|  |  |  |  |  |
| *Sr53* | 5Mg | BE442814 - BE442600 | 246821529 - 383441731 | AegTA2899_5M01G00000162500 |
|  |  |  |  | AegTA2899_5M01G00000164400 |
|  |  |  |  | AegTA2899_5M01G00000171100 |
|  |  |  |  | AegTA2899_5M01G00000176400 |
|  |  |  |  | AegTA2899_5M01G00000185100 |
|  |  |  |  | AegTA2899_5M01G00000190000 |
|  |  |  |  | AegTA2899_5M01G00000190100 |
|  |  |  |  | AegTA2899_5M01G00000194100 |
|  |  |  |  | AegTA2899_5M01G00000194300 |
|  |  |  |  | AegTA2899_5M01G00000194400 |
|  |  |  |  | AegTA2899_5M01G00000195200 |
|  |  |  |  | AegTA2899_5M01G00000196500 |
|  |  |  |  | AegTA2899_5M01G00000197900 |
|  |  |  |  | AegTA2899_5M01G00000198000 |
|  |  |  |  | AegTA2899_5M01G00000198200 |
|  |  |  |  | AegTA2899_5M01G00000198300 |
|  |  |  |  | AegTA2899_5M01G00000202900 |
|  |  |  |  | AegTA2899_5M01G00000203000 |
|  |  |  |  | AegTA2899_5M01G00000217100 |
|  |  |  |  | AegTA2899_5M01G00000218300 |
|  |  |  |  | AegTA2899_5M01G00000218400 |
|  |  |  |  | AegTA2899_5M01G00000218600 |
|  |  |  |  | AegTA2899_5M01G00000221900 |
|  |  |  |  | AegTA2899_5M01G00000232800 |
|  |  |  |  | AegTA2899_5M01G00000232900 |
|  |  |  |  | AegTA2899_5M01G00000241400 |
|  |  |  |  | AegTA2899_5M01G00000241500 |
|  |  |  |  | AegTA2899_5M01G00000245500 |
|  |  |  |  | AegTA2899_5M01G00000248800 |
|  |  |  |  | AegTA2899_5M01G00000251000 |
|  |  |  |  | AegTA2899_5M01G00000251100 |
|  |  |  |  | AegTA2899_5M01G00000260000 |
|  |  |  |  | AegTA2899_5M01G00000260200 |
|  |  |  |  | AegTA2899_5M01G00000264900 |
|  |  |  |  | AegTA2899_5M01G00000266500 |
|  |  |  |  | AegTA2899_5M01G00000269800 |
|  |  |  |  | AegTA2899_5M01G00000272900 |
|  |  |  |  | AegTA2899_5M01G00000273300 |
|  |  |  |  |  |

**Table S13:** Summary of RNA-seq data and expressed genes across different tissues in *Ae. geniculata*.

| **Tissue** | **Total reads** | **# reads pseudoaligned (%)** | **Genes expressed (TPM≥1)** |
| --- | --- | --- | --- |
| Flag leaf | 121,445,594 | 74.2 | 31,753 |
| Leaf sheath | 140,511,307 | 71.8 | 31,681 |
| Pre-anthesis spikes | 158,875,407 | 74.8 | 30,715 |
| Seedling leaf | 152,009,624 | 75.8 | 31,706 |
| Seedling root | 174,134,498 | 78.2 | 33,496 |
| Stem | 160,985,095 | 78.5 | 32,119 |
| Whole spike | 159,867,888 | 76.3 | 31,235 |

**Table S14:** Distribution of expressed genes across tissues. Gene is considered not expressed if it shows TPM < 1 in any given tissue.

| **Number of tissues where gene is expressed** | **Number of genes expressed** | **Proportion (%)** |
| --- | --- | --- |
| All | 25,469 | 70.61 |
| 6 | 3533 | 9.79 |
| 5 | 1985 | 5.50 |
| 4 | 1531 | 4.24 |
| 3 | 1243 | 3.45 |
| 2 | 1137 | 3.15 |
| 1* | 1172* | 3.25 |
| **Tissue-specific exp. of 1172 genes*** | **Gene count** | **Proportion (%) in tissue** |
| Seedling stage, leaf | 64 | 5.46 |
| Seedling stage, root | 590 | 50.34 |
| Flag leaf | 0 | 0.00 |
| Leaf sheath | 80 | 6.83 |
| Whole spike | 420 | 35.84 |
| Pre-anthesis spike | 18 | 1.54 |
| Stem | 0 | 0.00 |

* genes expressed in only one tissue (tissue-specific genes)

**Table S15.** Grouping genes based on the coefficient of variation (CV) in gene expression across seven tissues.

| **Category** | **N Genes** | **%** |
| --- | --- | --- |
| Highly variable | 7,606 | 21.1 |
| Intermediate | 19,476 | 54.0 |
| Conserved | 8,988 | 24.9 |

**Table S16.** Number of homoeologous genes from dyads classified according to gene expression CV across tissues.

|  | **U Conserved** | **U Highly variable** | **U Intermediate** | **U Not Expressed** |
| --- | --- | --- | --- | --- |
| **M Conserved** | 2763 (0.131) | 8 (0) | 799 (0.038) | 113 (0.005) |
| **M Highly variable** | 3 (0) | 1874 (0.089) | 389 (0.018) | 432 (0.02) |
| **M Intermediate** | 765 (0.036) | 462 (0.022) | 5199 (0.246) | 538 (0.025) |
| **M Not Expressed** | 72 (0.003) | 334 (0.016) | 411 (0.019) | 7005 (0.331) |

**Table S17:** Homoeolog expression classification classifications per tissue.

| **Tissue** | **Balanced** | **M dominant** | **U dominant** |
| --- | --- | --- | --- |
| Seedling_stage_leaf | 11155 | 1707 | 1300 |
| Seedling_stage_root | 11396 | 1592 | 1174 |
| Flag_leaf | 10619 | 1973 | 1570 |
| Leaf_sheth | 11085 | 1729 | 1348 |
| Whole_spike | 11421 | 1564 | 1177 |
| Pre_anthesis_spikes | 10874 | 1832 | 1456 |
| Stem | 10970 | 1779 | 1413 |
| Average number | 11074 | 1739 | 1348 |

**Table S18.** List of *Aegilops* species used for data curation.

| N | Species | Abbreviation | Ploidy | Subgenomes | Accessions, n |
| --- | --- | --- | --- | --- | --- |
| 1 | *Ae. bicornis* | Abic | 2n | S | 13 |
| 2 | *Ae. biuncialis* | Abiu | 4n | MU | 49 |
| 3 | *Ae. columnaris* | Acol | 4n | MU | 11 |
| 4 | *Ae. comosa* | Acom | 2n | M | 17 |
| 5 | *Ae. crassa* | Acra | 4n | DM | 31 |
| 6 | *Ae. cylindrica* | Acyl | 4n | CD | 78 |
| 7 | *Ae. geniculata* | Agen | 4n | MU | 141 |
| 8 | *Ae. juvenalis* | Ajuv | 6n | DMU | 9 |
| 9 | *Ae. kotschyi* | Akot | 4n | US | 26 |
| 10 | *Ae. longissima* | Alon | 2n | S | 12 |
| 11 | *Ae. markgrafii* | Amar | 2n | C | 21 |
| 12 | *Ae. neglecta* | Aneg | 4n | MU | 77 |
| 13 | *Ae. peregrina* | Aper | 4n | US | 31 |
| 14 | *Ae. searsii* | Ase | 2n | S | 21 |
| 15 | *Ae. sharonensis* | Asha | 2n | S | 9 |
| 16 | *Ae. speltoides* | Aspe | 2n | S | 96 |
| 17 | *Ae. tauschii* | Atau | 2n | D | 3 |
| 18 | *Ae. triuncialis* | Atri | 4n | CU | 197 |
| 19 | *Ae. umbellulata* | Aumb | 2n | U | 57 |
| 20 | *Ae. uniaristata* | Auni | 2n | N | 22 |
| 21 | *Ae. vavilovii* | Avav | 6n | DMS | 4 |
|  | Unknown | - | - | - | 11 |
|  | Total |  |  |  | 937 |

**Table S19.** General statistics* of the genomic variants in the 21 *Aegilops* species genotyped on the Aegilops_geniculata_ksu_v1.3 reference genome.

| Chromosome | Samples | Records | Indels | SNPs | Biallelic SNPs |
| --- | --- | --- | --- | --- | --- |
| Chr1M | 936 | 861,718 | 60,288 | 812,939 | 736,701 |
| Chr1U | 936 | 1,146,394 | 62,396 | 1,097,218 | 994,486 |
| Chr2M | 936 | 1,173,171 | 84,526 | 1,105,434 | 995,550 |
| Chr2U | 936 | 1,682,875 | 95,862 | 1,608,415 | 1,451,211 |
| Chr3M | 936 | 1,192,934 | 82,132 | 1,127,140 | 1,020,197 |
| Chr3U | 936 | 1,442,397 | 80,139 | 1,379,790 | 1,248,535 |
| Chr4M | 936 | 1,164,498 | 80,198 | 1,100,274 | 995,919 |
| Chr4U | 936 | 1,821,520 | 114,545 | 1,733,617 | 1,544,871 |
| Chr5M | 936 | 1,094,863 | 79,029 | 1,031,019 | 932,252 |
| Chr5U | 936 | 1,707,667 | 101,230 | 1,630,488 | 1,458,653 |
| Chr6M | 936 | 947,225 | 61,281 | 901,567 | 799,524 |
| Chr6U | 936 | 1,537,036 | 83,014 | 1,473,004 | 1,331,297 |
| Chr7M | 936 | 1,339,190 | 94,625 | 1,264,058 | 1,136,901 |
| Chr7U | 936 | 1,875,172 | 111,990 | 1,787,749 | 1,608,256 |
| ChrUN | 936 | 6,585 | 304 | 6,305 | 5,893 |
| Total | - | 18,993,245 | 1,191,559 | 18,059,017 | 16,260,246 |

* produced by bcftools stats (1.15.1+htslib-1.15.1) from raw combined vcf

**Table S20.** Data curation for species assignment using SNPs from the panel of 21 *Aegilops* species.

| Plant ID | Original label | SNP PCA |  | |
| --- | --- | --- | --- | --- |
|  |  | M | U | Status |
| TA1711 | *Ae. geniculata* | *Ae. triuncialis* | *Ae. triuncialis* | *exclude* |
| TA2043 | *Ae. geniculata* | *Ae. triuncialis* | *Ae. triuncialis* | *exclude* |
| TA11104 | *Ae. umbellulata* | *Ae. neglecta* | *Ae. neglecta* | *exclude* |
| TA1879 | *Ae. geniculata* | *Ae. geniculata* | *Ae. juvenalis* | *Ae. geniculata* |
| TA10847 | *Ae. geniculata* | *Ae. geniculata* | *Ae. geniculata* | *Ae. geniculata* |
| TA2221 | *Ae. geniculata* | *Ae. geniculata - Ae. triuncialis* | *Ae. geniculata - Ae. triuncialis* | *Ae. geniculata* |
| TA10002 | *Ae. geniculata* | *Ae. geniculata - Ae. triuncialis* | *Ae. geniculata - Ae. triuncialis* | *Ae. geniculata* |
| TA1854 | *Ae. umbellulata* | *Ae. speltoides- Ae. umbellulata* | *Ae. geniculata – Ae. speltoides* | *Ae. umbellulata* |
| TA1848 | *Ae. umbellulata* | *Ae. comosa -Ae. biuncialis* | *Ae. biuncialis - Ae. umbellulata* | *Ae. geniculata* |
| TA2633 | *Ae. umbellulata* | *-* | *Ae. biuncialis* | *Ae. umbellulata* |
| TA11097 | *Ae. umbellulata* | *-* | *Ae. umbellulata* | *Ae. umbellulata* |
| TA11085 | *Ae. peregrina* | *Ae. geniculata* | *Ae. geniculata* | *Ae. geniculata* |
| TA2231 | *Ae. triuncialis* | *Ae. geniculata* | *Ae. geniculata* | *Ae. geniculata* |
| TA2733 | *Ae. uniaristata* | *Ae. comosa* | *-* | *Ae. comosa* |

**Table S21.** List of *Ae. umbellulata,* *Ae. comosa* and *Ae. geniculata* accessions used for diversity analyses.

| **WGRC acc.** | **Geo. origin** | **Species** | **Genome** | **Acc. label** | **Lat.** | **Lon.** | **Elev. (m)** | **Country** | **Ae_gen pop. label** |
| --- | --- | --- | --- | --- | --- | --- | --- | --- | --- |
| TA1965 | AN | A.comosa | MM | AcomAN2n_TA1965subv | 40.93333 | 29.13333 | 30 | TUR |  |
| TA2171 | AN | A.comosa | MM | AcomAN2n_TA2171como | NA | NA | 30 | TUR |  |
| TA2731 | AN | A.comosa | MM | AcomAN2n_TA2731como | 38.38903 | 27.20352 | 235 | TUR |  |
| TA2732 | AN | A.comosa | MM | AcomAN2n_TA2732como | 38.38903 | 27.20352 | 235 | TUR |  |
| TA2735 | AN | A.comosa | MM | AcomAN2n_TA2735subv | 40.8167 | 29.5 | 105 | TUR |  |
| TA1967 | BA | A.comosa | MM | AcomBA2n_TA1967subv | 39.6 | 20.55 | 135 | GRC |  |
| TA2102 | BA | A.comosa | MM | AcomBA2n_TA2102como | 37.63544 | 23.15369 | 8 | GRC |  |
| TA2736 | BA | A.comosa | MM | AcomBA2n_TA2736subv | 37.4667 | 20.5 | 35 | GRC |  |
| TA2737 | BA | A.comosa | MM | AcomBA2n_TA2737subv | 39.63042 | 19.88628 | 1 | GRC |  |
| TA2756 | BA | A.comosa | MM | AcomBA2n_TA2756como | 39.83069 | 22.2583 | 200 | GRC |  |
| TA2757 | BA | A.comosa | MM | AcomBA2n_TA2757como | 39.38739 | 22.84195 | 250 | GRC |  |
| TA2758 | BA | A.comosa | MM | AcomBA2n_TA2758como | 37.963 | 23.81667 | 400 | GRC |  |
| TA2760 | BA | A.comosa | MM | AcomBA2n_TA2760como | 37.963 | 23.81667 | 210 | GRC |  |
| TA2761 | BA | A.comosa | MM | AcomBA2n_TA2761subv | 36.98333 | 22.33333 | 1080 | GRC |  |
| TA2733 | CA | A.comosa | MM | AcomCA2n_TA2733 | NA | NA | NA | RUS |  |
| TA2734 | CR | A.comosa | MM | AcomCR2n_TA2734subv | 35.50428 | 24.03809 | 45 | GRC |  |
| TA2104 | XX | A.comosa | MM | AcomXX2n_TA2104como | NA | NA | NA | GRC |  |
| PI551049 | BA | A.comosa | MM | AcomBA2n_PI551049ref1 | 37.6667 | 23.1 | 234 | GRC |  |
| TA10002 | AF | Ae.geniculata | MMUU | AgenAF4n_TA10002 | 32.56806 | -6.03083 | 470 | MAR | pop8 |
| TA10022 | AF | Ae.geniculata | MMUU | AgenAF4n_TA10022 | 33.29153 | -6.54365 | 720 | MAR | pop5 |
| TA10023 | AF | Ae.geniculata | MMUU | AgenAF4n_TA10023 | 33.53534 | -6.60846 | 305 | MAR | pop5 |
| TA2048 | AF | Ae.geniculata | MMUU | AgenAF4n_TA2048 | 33.73805 | -6.72089 | 135 | MAR | pop5 |
| TA2049 | AF | Ae.geniculata | MMUU | AgenAF4n_TA2049 | 33.53195 | -6.60622 | 490 | MAR | pop5 |
| TA2051 | AF | Ae.geniculata | MMUU | AgenAF4n_TA2051 | 33.42507 | -6.00177 | 1180 | MAR | pop5 |
| TA2184 | AF | Ae.geniculata | MMUU | AgenAF4n_TA2184 | 32.93944 | -5.6675 | 840 | MAR | pop5 |
| TA2220 | AF | Ae.geniculata | MMUU | AgenAF4n_TA2220 | 33.8362 | -6.18256 | 430 | MAR | pop5 |
| TA2223 | AF | Ae.geniculata | MMUU | AgenAF4n_TA2223 | 33.76659 | -5.48904 | 700 | MAR | pop5 |
| TA2235 | AF | Ae.geniculata | MMUU | AgenAF4n_TA2235 | 33.83848 | -4.63898 | 535 | MAR | pop5 |
| TA2238 | AF | Ae.geniculata | MMUU | AgenAF4n_TA2238 | 34.88635 | -2.01204 | 455 | MAR | pop5 |
| TA2239 | AF | Ae.geniculata | MMUU | AgenAF4n_TA2239 | 34.87016 | -2.42798 | 250 | MAR | pop5 |
| TA2241 | AF | Ae.geniculata | MMUU | AgenAF4n_TA2241 | 35.16371 | -3.09643 | 110 | MAR | pop5 |
| TA2245 | AF | Ae.geniculata | MMUU | AgenAF4n_TA2245 | 35.08339 | -4.02237 | 375 | MAR | pop5 |
| TA2251 | AF | Ae.geniculata | MMUU | AgenAF4n_TA2251 | 35.06485 | -5.12924 | 955 | MAR | pop5 |
| TA10021 | AF | Ae.geniculata | MMUU | AgenAF4n_TA10021 | 33.29153 | -6.54365 | 720 | MAR | pop7 |
| TA2231 | XX | Ae.geniculata | MMUU | Agen_TA2231 | 33.9069 | -4.98779 | 605 | MAR | pop5 |
| TA10003 | AF | Ae.geniculata | MMUU | AgenAF4n_TA10003 | 32.23909 | -6.12325 | 1060 | MAR | pop6 |
| TA10004 | AF | Ae.geniculata | MMUU | AgenAF4n_TA10004 | 32.23909 | -6.12325 | 1060 | MAR | pop6 |
| TA10005 | AF | Ae.geniculata | MMUU | AgenAF4n_TA10005 | 32.15948 | -6.35387 | 920 | MAR | pop6 |
| TA10006 | AF | Ae.geniculata | MMUU | AgenAF4n_TA10006 | 32.15948 | -6.35387 | 920 | MAR | pop6 |
| TA10007 | AF | Ae.geniculata | MMUU | AgenAF4n_TA10007 | 32.10643 | -6.45901 | NULL | MAR | pop6 |
| TA10008 | AF | Ae.geniculata | MMUU | AgenAF4n_TA10008 | 31.96614 | -6.5657 | 1380 | MAR | pop6 |
| TA10009 | AF | Ae.geniculata | MMUU | AgenAF4n_TA10009 | 31.87813 | -6.47033 | 1665 | MAR | pop6 |
| TA10011 | AF | Ae.geniculata | MMUU | AgenAF4n_TA10011 | 31.8633 | -6.9397 | 875 | MAR | pop6 |
| TA10012 | AF | Ae.geniculata | MMUU | AgenAF4n_TA10012 | 31.73333 | -7 | 935 | MAR | pop6 |
| TA10034 | AF | Ae.geniculata | MMUU | AgenAF4n_TA10034 | 31.1235 | -7.59945 | 1900 | MAR | pop7 |
| TA10035 | AF | Ae.geniculata | MMUU | AgenAF4n_TA10035 | 31.11152 | -7.59922 | 1925 | MAR | pop7 |
| TA10014 | AF | Ae.geniculata | MMUU | AgenAF4n_TA10014 | 31.73333 | -7 | 935 | MAR | pop6 |
| TA10015 | AF | Ae.geniculata | MMUU | AgenAF4n_TA10015 | 31.64505 | -7.26643 | 875 | MAR | pop6 |
| TA10018 | AF | Ae.geniculata | MMUU | AgenAF4n_TA10018 | 32.0152 | -6.7189 | 780 | MAR | pop6 |
| TA10019 | AF | Ae.geniculata | MMUU | AgenAF4n_TA10019 | 32.20709 | -6.53831 | 600 | MAR | pop6 |
| TA10040 | AF | Ae.geniculata | MMUU | AgenAF4n_TA10040 | 30.73387 | -9.82796 | 150 | MAR | pop7 |
| TA10041 | AF | Ae.geniculata | MMUU | AgenAF4n_TA10041 | 30.89162 | -9.73972 | 345 | MAR | pop4 |
| TA10042 | AF | Ae.geniculata | MMUU | AgenAF4n_TA10042 | 31.20341 | -9.70642 | 295 | MAR | pop7 |
| TA10043 | AF | Ae.geniculata | MMUU | AgenAF4n_TA10043 | 31.57503 | -9.67072 | 150 | MAR | pop4 |
| TA2040 | AF | Ae.geniculata | MMUU | AgenAF4n_TA2040 | 35.49869 | -5.77606 | 85 | MAR | pop4 |
| TA2041 | AF | Ae.geniculata | MMUU | AgenAF4n_TA2041 | 35.25123 | -6.08917 | 195 | MAR | pop4 |
| TA2042 | AF | Ae.geniculata | MMUU | AgenAF4n_TA2042 | 35.25123 | -6.08917 | 195 | MAR | pop2a |
| TA10020 | AF | Ae.geniculata | MMUU | AgenAF4n_TA10020 | 32.33704 | -6.36246 | 515 | MAR | pop6 |
| TA10024 | AF | Ae.geniculata | MMUU | AgenAF4n_TA10024 | 33.56848 | -6.8914 | 390 | MAR | pop6 |
| TA10025 | AF | Ae.geniculata | MMUU | AgenAF4n_TA10025 | 31.37454 | -7.79058 | 865 | MAR | pop6 |
| TA10026 | AF | Ae.geniculata | MMUU | AgenAF4n_TA10026 | 31.37454 | -7.79058 | 865 | MAR | pop6 |
| TA10027 | AF | Ae.geniculata | MMUU | AgenAF4n_TA10027 | 31.20267 | -7.85856 | 2625 | MAR | pop6 |
| TA10029 | AF | Ae.geniculata | MMUU | AgenAF4n_TA10029 | 31.53407 | -7.4889 | 970 | MAR | pop6 |
| TA10030 | AF | Ae.geniculata | MMUU | AgenAF4n_TA10030 | 31.37291 | -7.40281 | 1500 | MAR | pop6 |
| TA2054 | AF | Ae.geniculata | MMUU | AgenAF4n_TA2054 | 33.15868 | -5.84559 | 1175 | MAR | pop7 |
| TA10031 | AF | Ae.geniculata | MMUU | AgenAF4n_TA10031 | 31.15722 | -7.46315 | 1720 | MAR | pop6 |
| TA10032 | AF | Ae.geniculata | MMUU | AgenAF4n_TA10032 | 31.15722 | -7.46315 | 1720 | MAR | pop6 |
| TA10036 | AF | Ae.geniculata | MMUU | AgenAF4n_TA10036 | 30.93612 | -8.26987 | 1350 | MAR | pop6 |
| TA10037 | AF | Ae.geniculata | MMUU | AgenAF4n_TA10037 | 30.98195 | -8.22825 | 2092 | MAR | pop6 |
| TA10038 | AF | Ae.geniculata | MMUU | AgenAF4n_TA10038 | 30.98195 | -8.22825 | 2092 | MAR | pop6 |
| TA10039 | AF | Ae.geniculata | MMUU | AgenAF4n_TA10039 | 31.25 | -7.98333 | 1040 | MAR | pop6 |
| TA2063 | AF | Ae.geniculata | MMUU | AgenAF4n_TA2063 | 33.46582 | -5.1622 | 1615 | MAR | pop7 |
| TA2044 | AF | Ae.geniculata | MMUU | AgenAF4n_TA2044 | 31.8633 | -6.9397 | 875 | MAR | pop6 |
| TA2045 | AF | Ae.geniculata | MMUU | AgenAF4n_TA2045 | 32.93944 | -5.6675 | 840 | MAR | pop6 |
| TA2046 | AF | Ae.geniculata | MMUU | AgenAF4n_TA2046 | 32.71278 | -5.80417 | 725 | MAR | pop6 |
| TA2070 | AF | Ae.geniculata | MMUU | AgenAF4n_TA2070 | 32.71667 | -5.1 | 1510 | MAR | pop8 |
| TA2050 | AF | Ae.geniculata | MMUU | AgenAF4n_TA2050 | 33.66667 | -6.31667 | 220 | MAR | pop6 |
| TA2056 | AF | Ae.geniculata | MMUU | AgenAF4n_TA2056 | 32.93944 | -5.6675 | 840 | MAR | pop6 |
| TA2058 | AF | Ae.geniculata | MMUU | AgenAF4n_TA2058 | 32.93944 | -5.6675 | 840 | MAR | pop6 |
| TA2059 | AF | Ae.geniculata | MMUU | AgenAF4n_TA2059 | 32.93944 | -5.6675 | 840 | MAR | pop6 |
| TA2060 | AF | Ae.geniculata | MMUU | AgenAF4n_TA2060 | 33.28752 | -5.33901 | 1460 | MAR | pop6 |
| TA2061 | AF | Ae.geniculata | MMUU | AgenAF4n_TA2061 | 33.44145 | -5.2248 | 1400 | MAR | pop6 |
| TA2188 | AF | Ae.geniculata | MMUU | AgenAF4n_TA2188 | 33.53381 | -5.46673 | 900 | MAR | pop7 |
| TA2062 | AF | Ae.geniculata | MMUU | AgenAF4n_TA2062 | 33.46582 | -5.1622 | 1615 | MAR | pop6 |
| TA2066 | AF | Ae.geniculata | MMUU | AgenAF4n_TA2066 | 32.92922 | -5.05206 | 1745 | MAR | pop6 |
| TA2068 | AF | Ae.geniculata | MMUU | AgenAF4n_TA2068 | 32.68042 | -4.73064 | 1470 | MAR | pop6 |
| TA2069 | AF | Ae.geniculata | MMUU | AgenAF4n_TA2069 | 32.71667 | -5.1 | 1510 | MAR | pop6 |
| TA2221 | AF | Ae.geniculata | MMUU | AgenAF4n_TA2221 | 33.92399 | -5.38742 | 535 | MAR | pop8 |
| TA2071 | AF | Ae.geniculata | MMUU | AgenAF4n_TA2071 | 32.73333 | -5.51667 | 1380 | MAR | pop6 |
| TA2072 | AF | Ae.geniculata | MMUU | AgenAF4n_TA2072 | 32.73333 | -5.51667 | 1380 | MAR | pop6 |
| TA2183 | AF | Ae.geniculata | MMUU | AgenAF4n_TA2183 | 32.93944 | -5.6675 | 840 | MAR | pop6 |
| TA2185 | AF | Ae.geniculata | MMUU | AgenAF4n_TA2185 | 33.16667 | -5.56667 | 1115 | MAR | pop6 |
| TA2187 | AF | Ae.geniculata | MMUU | AgenAF4n_TA2187 | 33.16667 | -5.56667 | 1115 | MAR | pop6 |
| TA2189 | AF | Ae.geniculata | MMUU | AgenAF4n_TA2189 | 32.56806 | -6.03083 | 470 | MAR | pop6 |
| TA2191 | AF | Ae.geniculata | MMUU | AgenAF4n_TA2191 | 32.56806 | -6.03083 | 470 | MAR | pop6 |
| TA2219 | AF | Ae.geniculata | MMUU | AgenAF4n_TA2219 | 34.03104 | -6.66252 | 155 | MAR | pop6 |
| TA2222 | AF | Ae.geniculata | MMUU | AgenAF4n_TA2222 | 33.97831 | -5.24331 | 395 | MAR | pop6 |
| TA2225 | AF | Ae.geniculata | MMUU | AgenAF4n_TA2225 | 33.68892 | -5.37231 | 990 | MAR | pop6 |
| TA2227 | AF | Ae.geniculata | MMUU | AgenAF4n_TA2227 | 33.54254 | -5.32013 | 1370 | MAR | pop6 |
| TA2242 | AF | Ae.geniculata | MMUU | AgenAF4n_TA2242 | 35.04855 | -2.96013 | 115 | MAR | pop4 |
| TA2232 | AF | Ae.geniculata | MMUU | AgenAF4n_TA2232 | 33.82921 | -4.84051 | 840 | MAR | pop6 |
| TA2237 | AF | Ae.geniculata | MMUU | AgenAF4n_TA2237 | 34.59902 | -2.01084 | 670 | MAR | pop6 |
| TA2240 | AF | Ae.geniculata | MMUU | AgenAF4n_TA2240 | 34.84142 | -2.41734 | 350 | MAR | pop6 |
| TA2253 | AF | Ae.geniculata | MMUU | AgenAF4n_TA2253 | 35.35994 | -5.36957 | 485 | MAR | pop2a |
| TA2255 | AF | Ae.geniculata | MMUU | AgenAF4n_TA2255 | 35.56128 | -5.44785 | 210 | MAR | pop4 |
| TA2256 | AF | Ae.geniculata | MMUU | AgenAF4n_TA2256 | 35.53646 | -5.72338 | 130 | MAR | pop4 |
| TA10849 | AN | Ae.geniculata | MMUU | AgenAN4n_TA10849 | 38.55 | 14.58333 | 120 | ITA | pop2a |
| TA1797 | AN | Ae.geniculata | MMUU | AgenAN4n_TA1797 | NA | NA | NA | TUR | pop2b |
| TA1798 | AN | Ae.geniculata | MMUU | AgenAN4n_TA1798 | 40.38333 | 26.61667 | 51 | TUR | pop4 |
| TA1799 | AN | Ae.geniculata | MMUU | AgenAN4n_TA1799 | 40.08333 | 27.56667 | 284 | TUR | pop4 |
| TA1801 | AN | Ae.geniculata | MMUU | AgenAN4n_TA1801 | 40.5232 | 29.31496 | 185 | TUR | pop3 |
| TA1802 | AN | Ae.geniculata | MMUU | AgenAN4n_TA1802 | 38.75355 | 26.96114 | 13 | TUR | pop2a |
| TA1803 | AN | Ae.geniculata | MMUU | AgenAN4n_TA1803 | 38.79376 | 26.9892 | 60 | TUR | pop2a |
| TA1806 | AN | Ae.geniculata | MMUU | AgenAN4n_TA1806 | 39.04839 | 27.09435 | 30 | TUR | pop2a |
| TA1807 | AN | Ae.geniculata | MMUU | AgenAN4n_TA1807 | 40.62073 | 28.95653 | 75 | TUR | pop3 |
| TA1812 | AN | Ae.geniculata | MMUU | AgenAN4n_TA1812 | 40.50861 | 30.17 | 25 | TUR | pop3 |
| TA1818 | AN | Ae.geniculata | MMUU | AgenAN4n_TA1818 | 41.09772 | 30.18356 | 50 | TUR | pop3 |
| TA1821 | AN | Ae.geniculata | MMUU | AgenAN4n_TA1821 | 39.47649 | 33.8475 | 895 | TUR | pop4 |
| TA1848 | AN | Ae.geniculata | MMUU | AgenAN4n_TA1848 | 36.5504 | 29.67165 | 1200 | TUR | pop8 |
| TA1997 | AN | Ae.geniculata | MMUU | AgenAN4n_TA1997 | 40.40147 | 27.83077 | 15 | TUR | pop4 |
| TA2147 | AN | Ae.geniculata | MMUU | AgenAN4n_TA2147 | 40.26931 | 28.05428 | 30 | TUR | pop4 |
| TA2647 | AN | Ae.geniculata | MMUU | AgenAN4n_TA2647 | 41.32191 | 34.8543 | 450 | TUR | pop3 |
| TA2648 | AN | Ae.geniculata | MMUU | AgenAN4n_TA2648 | NA | NA | NA | TUR | pop2a |
| TA10847 | BA | Ae.geniculata | MMUU | AgenBA4n_TA10847 | 41.43333 | 22.65 | 227 | SRB | pop3 |
| TA1702 | BA | Ae.geniculata | MMUU | AgenBA4n_TA1702 | NA | NA | NA | ROU | pop1 |
| TA1800 | BA | Ae.geniculata | MMUU | AgenBA4n_TA1800 | 41.4325 | 27.09306 | 51 | TUR | pop3 |
| TA1814 | BA | Ae.geniculata | MMUU | AgenBA4n_TA1814 | NA | NA | NA | ROU | pop1 |
| TA2786 | BA | Ae.geniculata | MMUU | AgenBA4n_TA2786 | 43.33479 | 17.81107 | 700 | BIH | pop3 |
| TA2787 | BA | Ae.geniculata | MMUU | AgenBA4n_TA2787 | 42.72684 | 17.99252 | 20 | HRV | pop3 |
| TA10850 | CR | Ae.geniculata | MMUU | AgenCR4n_TA10850 | 44.51167 | 33.83472 | 420 | UKR | pop3 |
| TA11109 | IT | Ae.geniculata | MMUU | AgenIT4n_TA11109 | 40.10746 | 18.51988 | 13 | ITA | pop3 |
| TA1813 | IT | Ae.geniculata | MMUU | AgenIT4n_TA1813 | NA | NA | NA | ITA | pop1 |
| TA1701 | ME | Ae.geniculata | MMUU | AgenME4n_TA1701 | 36.60009 | 36.19686 | 50 | TUR | pop2b |
| TA1710 | ME | Ae.geniculata | MMUU | AgenME4n_TA1710 | 36.54054 | 36.15257 | 250 | TUR | pop2b |
| TA1721 | ME | Ae.geniculata | MMUU | AgenME4n_TA1721 | 36.1473 | 36.05919 | 180 | TUR | pop2b |
| TA1804 | ME | Ae.geniculata | MMUU | AgenME4n_TA1804 | 37.19096 | 36.38123 | 400 | TUR | pop2b |
| TA1805 | ME | Ae.geniculata | MMUU | AgenME4n_TA1805 | 37.10338 | 37.44141 | 1000 | TUR | pop2b |
| TA1808 | ME | Ae.geniculata | MMUU | AgenME4n_TA1808 | 37.03545 | 37.71057 | 650 | TUR | pop2a |
| TA1809 | ME | Ae.geniculata | MMUU | AgenME4n_TA1809 | 40.44264 | 30.05104 | 50 | TUR | pop3 |
| TA1810 | ME | Ae.geniculata | MMUU | AgenME4n_TA1810 | 31.79327 | 35.24364 | 815 | ISR | pop2b |
| TA1811 | ME | Ae.geniculata | MMUU | AgenME4n_TA1811 | 31.77755 | 35.18944 | 787 | ISR | pop2a |
| TA1879 | ME | Ae.geniculata | MMUU | AgenME4n_TA1879 | 32.0881 | 35.71415 | 970 | JOR | pop8 |
| TA2145 | ME | Ae.geniculata | MMUU | AgenME4n_TA2145 | 36.81478 | 36.93054 | 500 | TUR | pop2b |
| TA2146 | ME | Ae.geniculata | MMUU | AgenME4n_TA2146 | 36.81478 | 36.93054 | 500 | TUR | pop2b |
| TA2650 | ME | Ae.geniculata | MMUU | AgenME4n_TA2650 | 36.63972 | 38.03861 | 350 | SYR | pop2b |
| TA2651 | ME | Ae.geniculata | MMUU | AgenME4n_TA2651 | 36.19444 | 36.75 | 450 | SYR | pop2a |
| TA2652 | ME | Ae.geniculata | MMUU | AgenME4n_TA2652 | 36.06556 | 36.865 | 400 | SYR | pop2b |
| TA2653 | ME | Ae.geniculata | MMUU | AgenME4n_TA2653ovat | 36.68333 | 37 | 600 | SYR | pop2b |
| TA2654 | ME | Ae.geniculata | MMUU | AgenME4n_TA2654 | 32.1 | 35.7 | 800 | JOR | pop2a |
| TA10044 | XX | Ae.geniculata | MMUU | AgenXX4n_TA10044 | NA | NA | NA | MAR | pop4 |
| TA10437 | XX | Ae.geniculata | MMUU | AgenXX4n_TA10437 | NA | NA | NA | Kosovo | pop1 |
| TA11027 | XX | Ae.geniculata | MMUU | AgenXX4n_TA11027 | NA | NA | NA | XKX | pop1 |
| TA11028 | XX | Ae.geniculata | MMUU | AgenXX4n_TA11028glob | NA | NA | NA | XKX | pop4 |
| TA2250 | AF | Ae.geniculata | MMUU | AgenAF4n_TA2250 | 35.03562 | -5.02213 | 1235 | MAR | pop6 |
| TA1816 | XX | Ae.geniculata | MMUU | AgenXX4n_TA1816 | NA | NA | NA | CHE | pop1 |
| TA1817 | XX | Ae.geniculata | MMUU | AgenXX4n_TA1817 | NA | NA | NA | Kosovo | pop4 |
| TA1819 | XX | Ae.geniculata | MMUU | AgenXX4n_TA1819vulg | NA | NA | NA | JPN | pop1 |
| TA2899 | XX | Ae.geniculata | MMUU | AgenME4n_TA2899_1 | NA | NA | NA | ISR | pop2a |
| TA11085 | XX | Ae.geniculata | MMUU | Agen_TA11085 | 32.71667 | -5.1 | 1510 | MAR | pop7 |
| TA1815 | XX | Ae.geniculata | MMUU | AgenXX4n_TA1815 | NA | NA | NA | GBR | pop6 |
| TA10416 | AN | Ae.umbellulata | UU | AumbAN2n_TA10416 | NA | NA | NA | TUR |  |
| TA11100 | AN | Ae.umbellulata | UU | AumbAN2n_TA11100 | 38.81667 | 29.98333 | 1250 | TUR |  |
| TA11101 | AN | Ae.umbellulata | UU | AumbAN2n_TA11101 | 39.11667 | 27.18333 | 50 | TUR |  |
| TA11102 | AN | Ae.umbellulata | UU | AumbAN2n_TA11102 | 37.85 | 29.05 | 180 | TUR |  |
| TA11103 | AN | Ae.umbellulata | UU | AumbAN2n_TA11103 | 38.2 | 28.71667 | 305 | TUR |  |
| TA11105 | AN | Ae.umbellulata | UU | AumbAN2n_TA11105 | 39.58333 | 26.48333 | 420 | TUR |  |
| TA11106 | AN | Ae.umbellulata | UU | AumbAN2n_TA11106 | 39.58333 | 30.93333 | 900 | TUR |  |
| TA1822 | AN | Ae.umbellulata | UU | AumbAN2n_TA1822 | 37.78537 | 28.97987 | 690 | TUR |  |
| TA1823 | AN | Ae.umbellulata | UU | AumbAN2n_TA1823 | 37.28826 | 27.82562 | 450 | TUR |  |
| TA1826 | AN | Ae.umbellulata | UU | AumbAN2n_TA1826 | 39.29605 | 31.44287 | 845 | TUR |  |
| TA1827 | AN | Ae.umbellulata | UU | AumbAN2n_TA1827 | 37.96152 | 28.90778 | 150 | TUR |  |
| TA1834 | AN | Ae.umbellulata | UU | AumbAN2n_TA1834typi | 37.72294 | 30.2948 | 960 | TUR |  |
| TA1849 | AN | Ae.umbellulata | UU | AumbAN2n_TA1849typi | 37.4288 | 34.8785 | 785 | TUR |  |
| TA1852 | AN | Ae.umbellulata | UU | AumbAN2n_TA1852 | 37.03435 | 30.40878 | 500 | TUR |  |
| TA2137 | AN | Ae.umbellulata | UU | AumbAN2n_TA2137 | 36.33642 | 29.32663 | 200 | TUR |  |
| TA2139 | AN | Ae.umbellulata | UU | AumbAN2n_TA2139 | 36.26545 | 29.41452 | 175 | TUR |  |
| TA2165 | AN | Ae.umbellulata | UU | AumbAN2n_TA2165 | 39.04839 | 27.09435 | 30 | TUR |  |
| TA2628 | AN | Ae.umbellulata | UU | AumbAN2n_TA2628 | 0 | 0 | 30 | TUR |  |
| TA2629 | AN | Ae.umbellulata | UU | AumbAN2n_TA2629 | 39.04839 | 27.09435 | 30 | TUR |  |
| TA2630 | AN | Ae.umbellulata | UU | AumbAN2n_TA2630 | 39.66667 | 27.68333 | 200 | TUR |  |
| TA2632 | AN | Ae.umbellulata | UU | AumbAN2n_TA2632 | 37.88231 | 32.42014 | 1125 | TUR |  |
| TA2636 | AN | Ae.umbellulata | UU | AumbAN2n_TA2636 | 37.51762 | 37.71074 | 820 | TUR |  |
| TA2638 | AN | Ae.umbellulata | UU | AumbAN2n_TA2638 | 38.1016 | 28.83705 | 275 | TUR |  |
| TA2639 | AN | Ae.umbellulata | UU | AumbAN2n_TA2639 | 39.11674 | 27.18292 | 60 | TUR |  |
| TA2640 | AN | Ae.umbellulata | UU | AumbAN2n_TA2640 | 39.04839 | 27.09435 | 30 | TUR |  |
| TA2641 | AN | Ae.umbellulata | UU | AumbAN2n_TA2641 | 39.64324 | 27.03873 | 20 | TUR |  |
| TA2642 | AN | Ae.umbellulata | UU | AumbAN2n_TA2642 | 37.30628 | 27.9911 | 420 | TUR |  |
| TA2643 | AN | Ae.umbellulata | UU | AumbAN2n_TA2643 | 36.78014 | 28.01788 | 25 | TUR |  |
| TA2644 | AN | Ae.umbellulata | UU | AumbAN2n_TA2644 | 36.66539 | 29.92161 | 190 | TUR |  |
| TA2649 | AN | Ae.umbellulata | UU | AumbAN2n_TA2649 | 37.28826 | 27.82562 | 400 | TUR |  |
| TA10835 | CA | Ae.umbellulata | UU | AumbCA2n_TA10835 | 41.43139 | 46.65167 | 319 | AZE |  |
| TA10998 | CA | Ae.umbellulata | UU | AumbCA2n_TA10998 | 40.62542 | 48.24912 | 334 | AZE |  |
| TA10999 | CA | Ae.umbellulata | UU | AumbCA2n_TA10999 | 40.62528 | 48.24938 | 328 | AZE |  |
| TA1820 | CA | Ae.umbellulata | UU | AumbCA2n_TA1820 | 41.43139 | 46.65167 | 1830 | IRN |  |
| TA11097 | ME | Ae.umbellulata | UU | AumbME2n_TA11097 | 36.2514 | 36.7944 | 300 | SYR |  |
| TA11098 | ME | Ae.umbellulata | UU | AumbME2n_TA11098 | 38.38333 | 40.08333 | 925 | TUR |  |
| TA11099 | ME | Ae.umbellulata | UU | AumbME2n_TA11099 | 37.75 | 40.1 | 1000 | TUR |  |
| TA1825 | ME | Ae.umbellulata | UU | AumbME2n_TA1825 | 39.12154 | 40.83618 | 1200 | TUR |  |
| TA1828 | ME | Ae.umbellulata | UU | AumbME2n_TA1828 | 35.67515 | 45.53284 | 1300 | IRQ |  |
| TA1829 | ME | Ae.umbellulata | UU | AumbME2n_TA1829 | 36.12013 | 50.41077 | 1372 | IRN |  |
| TA1830 | ME | Ae.umbellulata | UU | AumbME2n_TA1830 | 32.61667 | 48.28333 | 366 | IRN |  |
| TA1831 | ME | Ae.umbellulata | UU | AumbME2n_TA1831 | 29.55 | 51.81667 | 1220 | IRN |  |
| TA1832 | ME | Ae.umbellulata | UU | AumbME2n_TA1832 | 29.72028 | 52.58667 | 1525 | IRN |  |
| TA1833 | ME | Ae.umbellulata | UU | AumbME2n_TA1833 | 29.63333 | 52.56667 | 1610 | IRN |  |
| TA1835 | ME | Ae.umbellulata | UU | AumbME2n_TA1835 | 36.4 | 44.13333 | 830 | IRQ |  |
| TA1850 | ME | Ae.umbellulata | UU | AumbME2n_TA1850typi | 33.43173 | 36.01799 | 820 | SYR |  |
| TA1855 | ME | Ae.umbellulata | UU | AumbME2n_TA1855 | 37.75172 | 37.85614 | 670 | TUR |  |
| TA2627 | ME | Ae.umbellulata | UU | AumbME2n_TA2627 | 37.48358 | 37.04315 | 515 | TUR |  |
| TA2633 | ME | Ae.umbellulata | UU | AumbME2n_TA2633 | 36.1133 | 36.5114 | 300 | SYR |  |
| TA2634 | ME | Ae.umbellulata | UU | AumbME2n_TA2634 | 37.2347 | 37.51007 | 800 | TUR |  |
| TA2635 | ME | Ae.umbellulata | UU | AumbME2n_TA2635 | 37.33686 | 37.56603 | 450 | TUR |  |
| TA2637 | ME | Ae.umbellulata | UU | AumbME2n_TA2637 | 37.63762 | 37.77649 | 900 | TUR |  |
| TA2800 | ME | Ae.umbellulata | UU | AumbME2n_TA2800 | 37.96098 | 40.32532 | 650 | TUR |  |
| TA1854 | ME | Ae.umbellulata | UU | AumbME4n_TA1854 | 37.52253 | 38.89195 | 625 | TUR |  |
| TA1824 | XX | Ae.umbellulata | UU | AumbXX2n_TA1824 | NA | NA | NA | AFG |  |
| TA1851 | XX | Ae.umbellulata | UU | AumbXX2n_TA1851 | NA | NA | NA | GBR |  |

**Table S22.** General statistics of the SNPs called in the M and U genomes by mapping reads from *Ae. umbellulata,* *Ae. comosa* and *Ae. geniculata* to Aegilops_geniculata_ksu_v1.3 reference genome.

| PLINK filters | | Input BED | | | Filtered BED | | | Filtered & LD pruned BED | | |
| --- | --- | --- | --- | --- | --- | --- | --- | --- | --- | --- |
| --maf | --geno | TGR | SNPs | Inds | TGR | SNPs | Inds | TGR | SNPs | Inds |
| M |  |  |  |  |  |  |  |  |  |  |
| 0.05 | 0.10 | 0.13 | 6616475 | 157 | 0.94 | 5632 | 157 | 0.94 | 4116 | 157 |
| 0.05 | 0.20 | 0.13 | 6616475 | 157 | 0.89 | 11802 | 157 | 0.89 | 8720 | 157 |
| 0.05 | 0.30 | 0.13 | 6616475 | 157 | 0.84 | 17330 | 157 | 0.84 | 12586 | 157 |
| 0.05 | 0.40 | 0.13 | 6616475 | 157 | 0.80 | 22870 | 157 | 0.79 | 16275 | 157 |
| **0.05** | **0.50** | **0.13** | **6616475** | **157** | **0.74** | **29355** | **157** | **0.74** | **20149*** | **157** |
| 0.05 | 0.60 | 0.13 | 6616475 | 157 | 0.67 | 37895 | 157 | 0.68 | 24588 | 157 |
| 0.05 | 0.70 | 0.13 | 6616475 | 157 | 0.60 | 49563 | 157 | 0.61 | 29434 | 157 |
| U |  |  |  |  |  |  |  |  |  |  |
| 0.05 | 0.10 | 0.14 | 9636698 | 199 | 0.95 | 5469 | 199 | 0.95 | 4536 | 199 |
| 0.05 | 0.20 | 0.14 | 9636698 | 199 | 0.90 | 10006 | 199 | 0.90 | 8078 | 199 |
| 0.05 | 0.30 | 0.14 | 9636698 | 199 | 0.85 | 14859 | 199 | 0.85 | 11714 | 199 |
| 0.05 | 0.40 | 0.14 | 9636698 | 199 | 0.79 | 20489 | 199 | 0.80 | 15838 | 199 |
| **0.05** | **0.50** | **0.14** | **9636698** | **199** | **0.73** | **26886** | **199** | **0.74** | **20078*** | **199** |
| 0.05 | 0.60 | 0.14 | 9636698 | 199 | 0.66 | 35277 | 199 | 0.68 | 24995 | 199 |
| 0.05 | 0.70 | 0.14 | 9636698 | 199 | 0.58 | 48097 | 199 | 0.60 | 30817 | 199 |

*Selected for diversity analyses reported in the current study.

**Supplementary Figures**


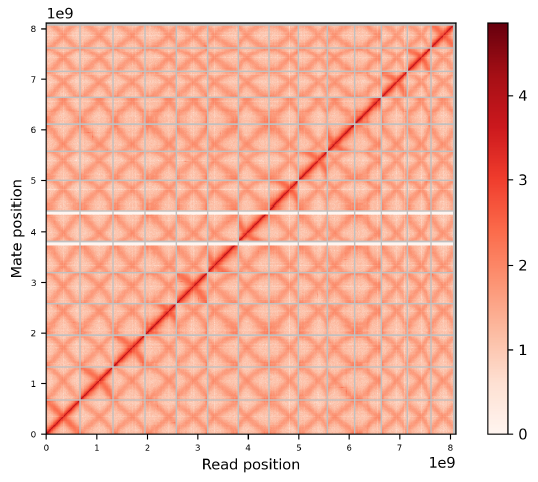


**Figure S1.** OmniC reads links density heatmap in the HiRise scaffolded *Ae. geniculata* pseudomolecules. The intensity of colors on the heatmap reflects the frequency of contacts on Hi-C map.

**Figure S2.** Heatmap of the depth of reads coverage derived from genomic DNA of the *T. aestivum* - *Ae. geniculata* disomic addition (DA) lines. The reads were aligned to the genomes of CS2.1 and *Ae. geniculata*. The depth was calculated for 50Mbp windows (colored verticals lines) and normalized to a median. The intensity of red and blue colors reflect increase or decrease in the depth of read coverage, respectively, relative to genome-wide median coverage shown in white color.


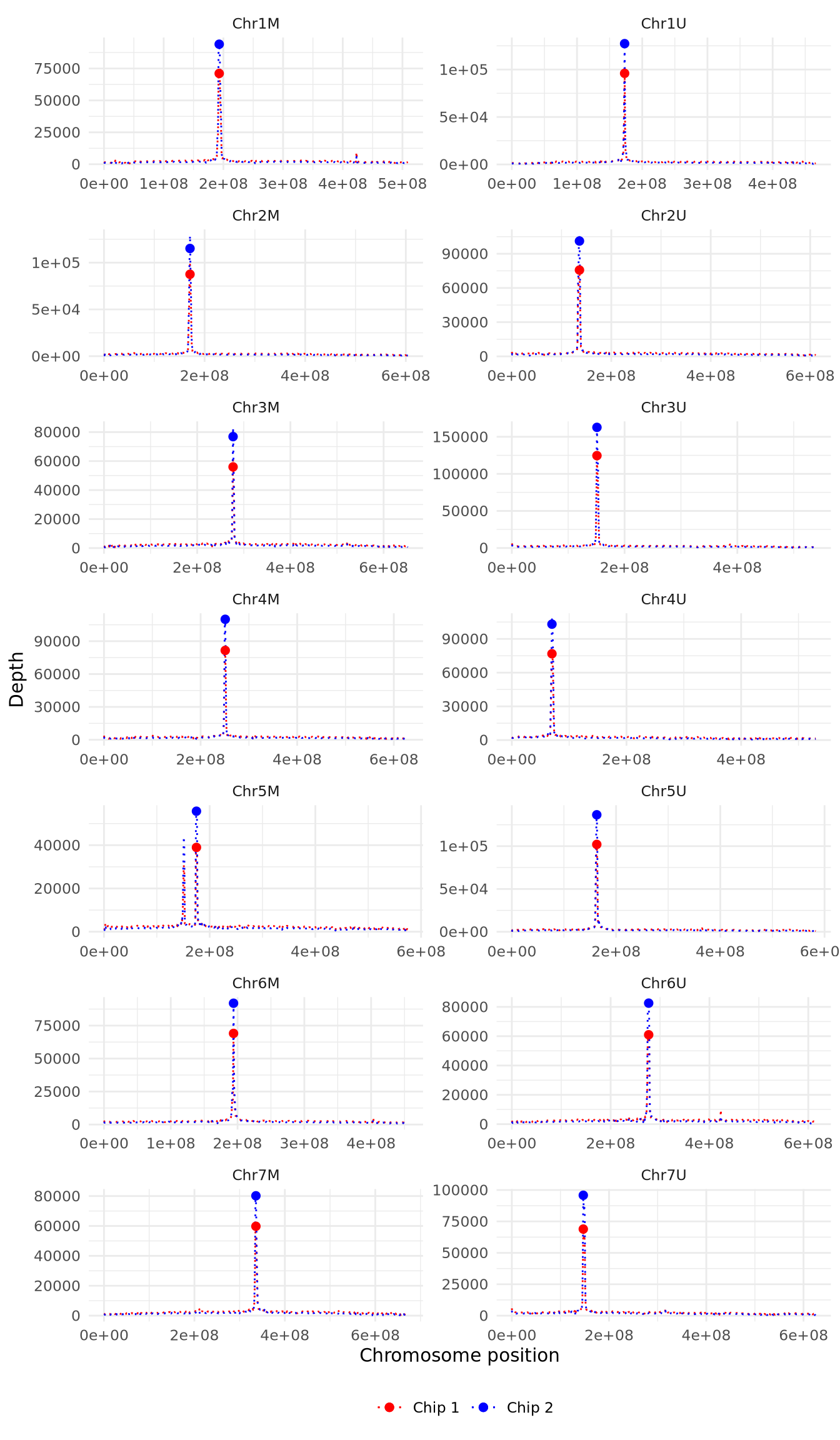


**Figure S3.** Centromere positions based on the CenH3 ChIP-seq data. The red and blue dashed lines correspond to results obtained using ChIP-seq biological replicates. The dots mark the summit of each peak.


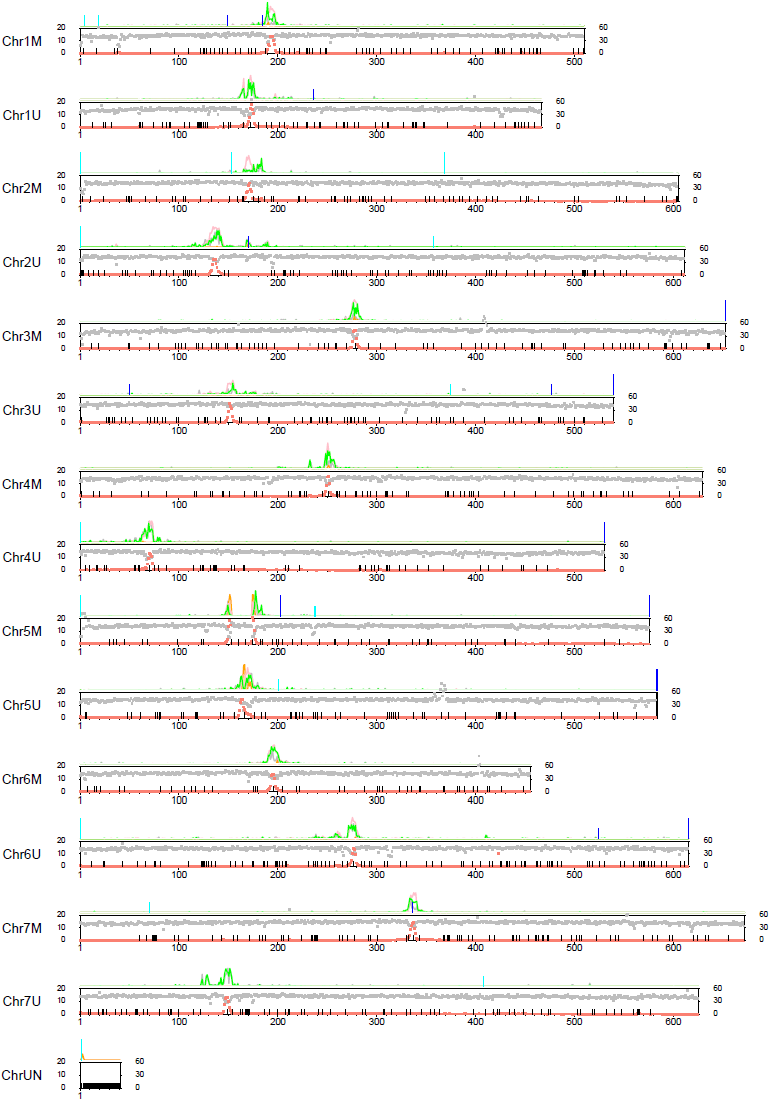


**Figure S4.** Ideogram of the Aegilops_geniculata_ksu_v1.3.fna assembly pseudomolecules with telomeric TTTAGGG (forward−blue/reverse complement−cyan, 10tandems−long/5tandems−short columns), centromeric CENH3 (orange), CRW2_LG_3L(pink), Quinta_LTRL3(green), Quinta_R5(grey) motifs density, mean depth of reads coverage (dot=1Mb window) for the back aligned TA2899 HiFi reads (gray), TA2899 CENH3 ChIP-seq reads (salmon red), and gaps between contigs (black columns). Length of the pseudomolecules given in Mbp in x-axis, the HiFi reads depth y-axis max is 20x, and for CENH3 ChIP-seq reads y axis max is 60x.


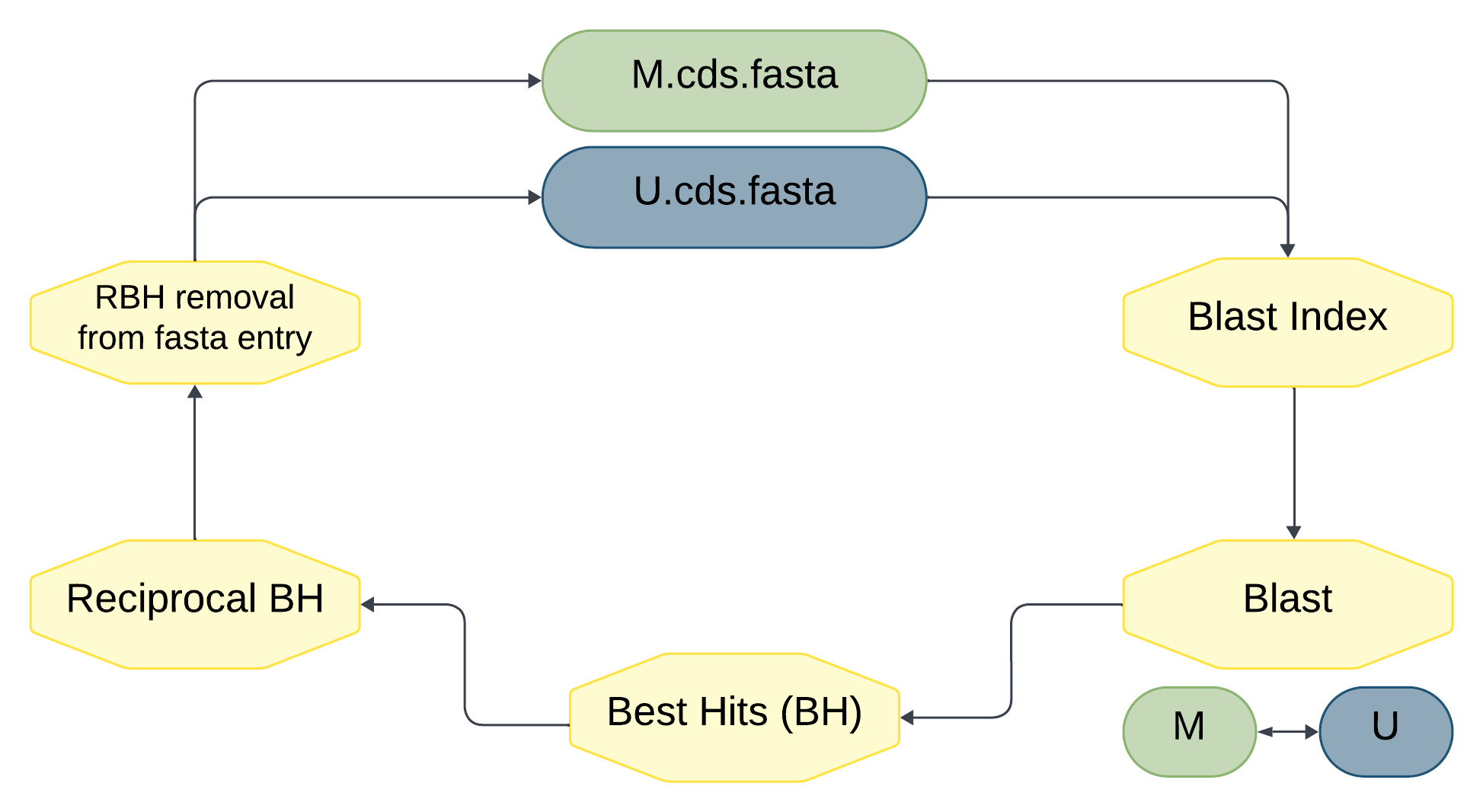


**Figure S5.** Detection of duplicated homoeologs and U^g^ and M^g^ genome-specific sets of genes by reciprocal best blast hit analysis.

**Figure S6.** Dot plots show alignment of U^g^ genome chromosomes with homoeologous chromosomes from M^g^ and wheat D genome.

**Figure S7.** Dot plots show alignment of 4U^g^ chromosome with chromosomes from the M^g^ genome.

**Figure S8.** Distribution of Homoeologous Expression Bias (HEB) values calculated using gene expression data from different tissues.

**Figure S9.** PCA analyses of *Aegilops* species in the diversity panel from the KSU WGRC collection. This analysis was used to exclude mis-classified accessions*.* Only those accessions that co-cluster with samples labeled as *Ae. geniculata*, *Ae. comosa* or *Ae. umbellulata* were retained.

**Figure S10.** PCA analyses of polyploids carrying the M and/or U genomes was aimed at evaluating their clustering patterns with the diploid genome donors *Ae. comosa* (M) and *Ae. umbellulata* (U).
